# Mixed-ligand complexes of paddlewheel dinuclear molybdenum as hydrodehalogenation catalysts for polyhaloalkanes†
†Electronic supplementary information (ESI) available: Experimental details for the synthesis and characterization of Mo_2_ complexes, kinetic analysis of the reactions, ^1^H NMR spectra of the catalytic reactions, identification of the products, CV of selected Mo_2_ complexes, and crystal data for **7a** (CCDC 1046579), **9a** (CCDC 1046580), **10a** (CCDC 1046581), and **11a** (CCDC 1046582). CCDC 1046579–1046582. For ESI and crystallographic data in CIF or other electronic format see DOI: 10.1039/c5sc00721f


**DOI:** 10.1039/c5sc00721f

**Published:** 2015-03-31

**Authors:** Hayato Tsurugi, Akio Hayakawa, Shun Kando, Yoshitaka Sugino, Kazushi Mashima

**Affiliations:** a Department of Chemistry , Graduate School of Engineering Science , Osaka University , CREST , Toyonaka , Osaka 560-8531 , Japan . Email: tsurugi@chem.es.osaka-u.ac.jp ; Email: mashima@chem.es.osaka-u.ac.jp

## Abstract

We developed a hydrodehalogenation reaction of polyhaloalkanes catalyzed by paddlewheel dimolybdenum complexes in combination with 1-methyl-3,6-bis(trimethylsilyl)-1,4-cyclohexadiene (MBTCD) as a non-toxic H-atom source as well as a salt-free reductant. A mixed-ligated dimolybdenum complex Mo_2_(OAc)_2_[CH(NAr)_2_]_2_ (**3a**, Ar = 4-MeOC_6_H_4_) having two acetates and two amidinates exhibited high catalytic activity in the presence of ^*n*^Bu_4_NCl, in which [^*n*^Bu_4_N]_2_[Mo_2_{CH(NAr)_2_}_2_Cl_4_] (**9a**), derived by treating **3a** with ClSiMe_3_ and ^*n*^Bu_4_NCl, was generated as a catalytically-active species in the hydrodehalogenation. All reaction processes, oxidation and reduction of the dimolybdenum complex, were clarified by control experiments, and the oxidized product, [^*n*^Bu_4_N][Mo_2_{CH(NAr)_2_}_2_Cl_4_] (**10a**), was characterized by EPR and X-ray diffraction studies. Kinetic analysis of the hydrodehalogenation reaction as well as a deuterium-labelling experiment using MBTCD-*d*_8_ suggested that the H-abstraction was the rate-determining step for the catalytic reaction.

## Abstract 2

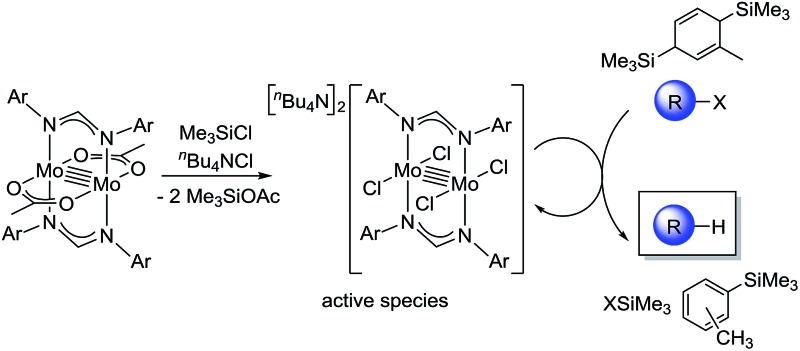
A mixed-ligated dimolybdenum complex Mo_2_(OAc)_2_[CH(NAr)_2_]_2_ in combination with 1-methyl-3,6-bis(trimethylsilyl)-1,4-cyclohexadiene and ^*n*^Bu_4_NCl exhibited high catalytic activity for hydrodehalogenation reactions.

## Introduction

**Fig. 1:**
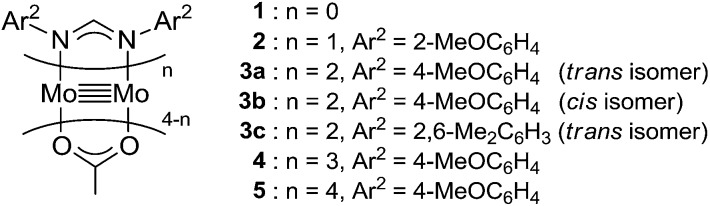
Paddlewheel dimolybdenum complexes **1–5**.

The M_2_L_4_-type paddlewheel dinuclear complexes having monoanionic bridging ligands comprise the simplest metal cluster motif, and intensive investigation has been focused on their structures, redox behaviors, and spectroscopic properties, as well as their catalytic applications.^1^–^3^ Dinuclear paddlewheel complexes of the late transition metals such as rhodium and ruthenium act as useful and versatile catalysts for cyclopropanation of olefins and for functionalizing C–H, O–H, N–H, and Si–H bonds *via* the generation of metal-carbene, [M_2_L_4_(=CR^1^R^2^)], metal-alkoxide, and -nitride species, [M_2_L_4_(Z)] (Z = OR, N).3c–3e,^4^–^6^ In all cases, because four supporting ligands tightly coordinate to the adjacent two metal centers while maintaining the dinuclear paddlewheel skeleton and the metal–metal bond, the architecture of the four supporting ligands was tunable to control redox behavior and catalytic performance of the paddlewheel complexes. In sharp contrast, few studies have examined the catalytic application of paddlewheel complexes of the early transition metals. As an example, Mo_2_(OAc)_4_ was used for an aza-Diels–Alder reaction of acyl hydrazones and dienes;^7^ however, the original paddlewheel structure was not maintained during the reaction. We and others have continued to investigate the stoichiometric and catalytic application of quadruply bonded M_2_ complexes of group 6 metals for organic radical generation by designing bridging ligands,^8^–^10^ and have achieved catalytic radical addition and polymerization reactions.^9^ During these transformations, the metal–metal bond responds to the one-electron redox processes without decomposition of the dinuclear structure. The structural stability of the dinuclear motif owes to both the surrounding four ligands and the metal–metal multiple bonds. In a further catalytic application of the M_2_ complexes, we used a cyclohexadiene derivative instead of α-olefins as a substrate for the organic radicals generated from polyhaloalkanes, leading to the formation of hydrodehalogenated products. Although hydrodehalogenation is one of the key reactions for decomposing environmentally unfriendly polyhaloalkanes, precious metal catalysts are often used.^11^ Herein, we report that paddlewheel Mo_2_ complexes, as shown in Fig. 1, act as catalysts for hydrodehalogenation reactions of polyhaloalkanes upon combination with 1-methyl-3,6-bis(trimethylsilyl)-1,4-cyclohexadiene (MBTCD)^12^ as an H-atom donor, and reveal the mechanism and actual active species in this catalytic process.

## Results and discussion

We began by searching for the best paddlewheel Mo_2_ catalyst among the homoleptic and mixed-ligand complexes listed in Fig. 1 for the hydrodehalogenation reaction of 1,1,1,3-tetrachloropropane, as a model substrate, in CD_3_CN in the presence of 1.2 equiv. of MBTCD to give 1,1,3-trichloropropane (Table 1).^13^ Simple homoleptic complexes **1** and **5** exhibited very low activities (runs 1 and 7), while mixed-ligand complexes of dimolybdenum bearing both acetate and amidinate ligands exhibited moderate catalytic activities (runs 2, 3, and 6). The stereochemistry of the mixed-ligand complexes was a key factor: *trans*-Mo_2_(OAc)_2_[CH(NAr)_2_]_2_ (Ar = 4-MeOC_6_H_4_) (**3a**) afforded the hydrodehalogenated product in 64% yield, whereas *cis*-Mo_2_(OAc)_2_[CH(NAr)_2_]_2_ (**3b**) afforded only a very low yield of the dehalogenated product (run 4). In addition, a *trans*-arranged mixed-ligated complex **3c** having bulky 2,6-dimethylphenyl groups at the nitrogen atoms of the amidinate ligand exhibited almost no activity, probably because the substrate approach was sterically prevented (run 5). Accordingly, we examined the solvent and additive effects using **3a** as the catalyst. In THF-*d*_8_ and C_6_D_6_, the yield of 1,1,3-trichloropropane decreased (runs 8 and 9). Positive additive effects of ^*n*^Bu_4_NCl were observed: addition of ^*n*^Bu_4_NCl (10 mol%) to the reaction mixture in acetonitrile led to the formation of 1,1,3-trichloropropane in 84% yield (run 10). In the blank reaction without MBTCD, no reaction proceeded since MBTCD acts as the H-donor and reductant for the catalytic reaction. When we used HSiEt_3_ as a commercially available H-donor instead of MBTCD, the yield decreased (11%), probably due to the inefficient reducing ability of the *in situ* generated intermediate [Mo_2_]^5+^ species (*vide infra*).

**Table 1:** Hydrodehalogenation reaction of 1,1,1,3-tetrachloropropane catalyzed by Mo_2_ paddlewheel complexes

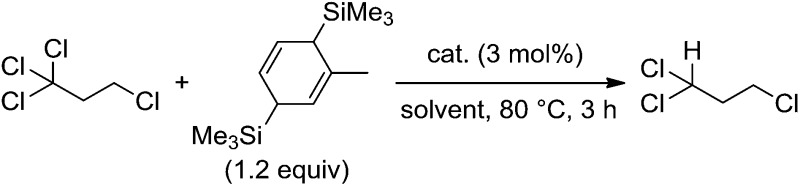				
Run	Cat.	Solvent	Additive	Yield^*a*^ (%)
1	**1**	CD_3_CN	—	4
2	**2**	CD_3_CN	—	47
3	**3a**	CD_3_CN	—	64
4	**3b**	CD_3_CN	—	9
5	**3c**	CD_3_CN	—	1
6	**4**	CD_3_CN	—	31
7	**5**	CD_3_CN	—	<1
8	**3a**	THF-*d*_8_	—	59
9	**3a**	C_6_D_6_	—	42
10^*b*^	**3a**	CD_3_CN	^*n*^Bu_4_NCl	84 (73)
11	**7a**	CD_3_CN	—	71
12^*b*^	**7a**	CD_3_CN	^*n*^Bu_4_NCl	85
13^*b*^	**8a**	CD_3_CN	^*n*^Bu_4_NCl	32
14	**9a**	CD_3_CN	—	85

^*a*^Determined using ^1^H NMR measurements. Yield in parentheses was the isolated yield.

^*b*^ ^*n*^Bu_4_NCl (10 mol%) was added to the reaction mixture.

**Scheme 1:**
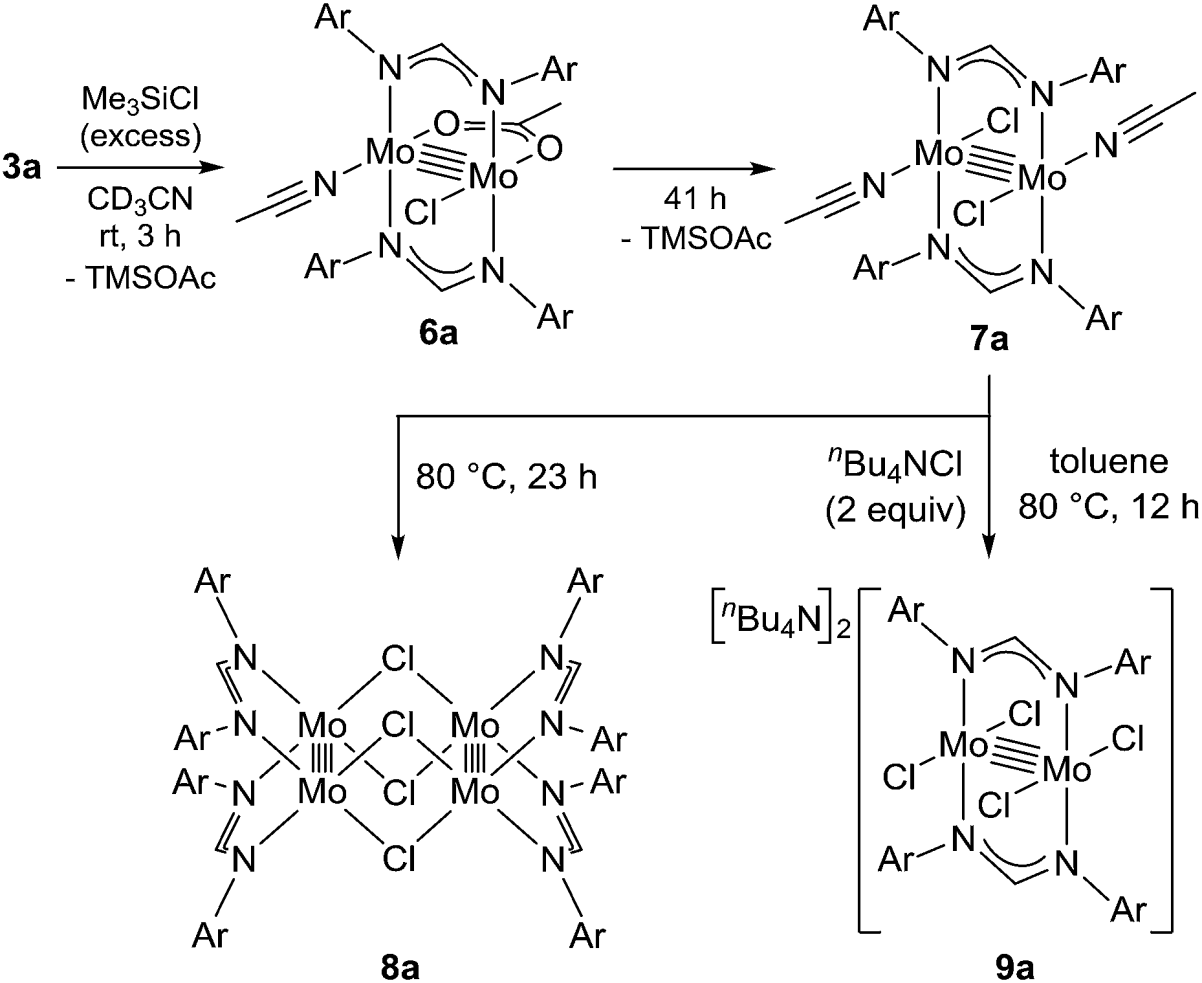
Ligand substitution and dimerization reactions of **3a** by the addition of Me_3_SiCl.

**Fig. 2:**
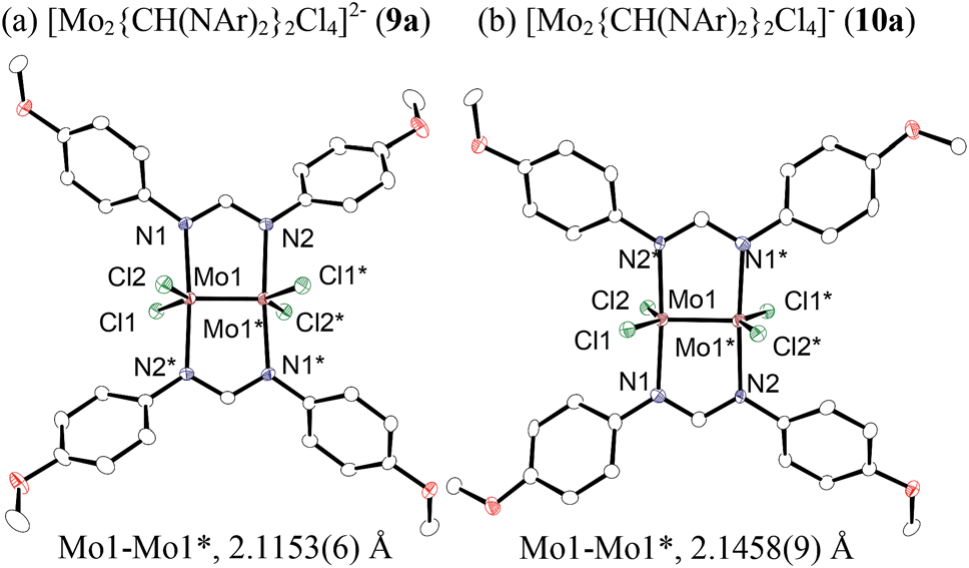
ORTEP drawing of the molecular structure for dimolybdenum complexes (a) **9a** and (b) **10a**. Hydrogen atoms and the cationic part are omitted for clarity.

**Fig. 3:**
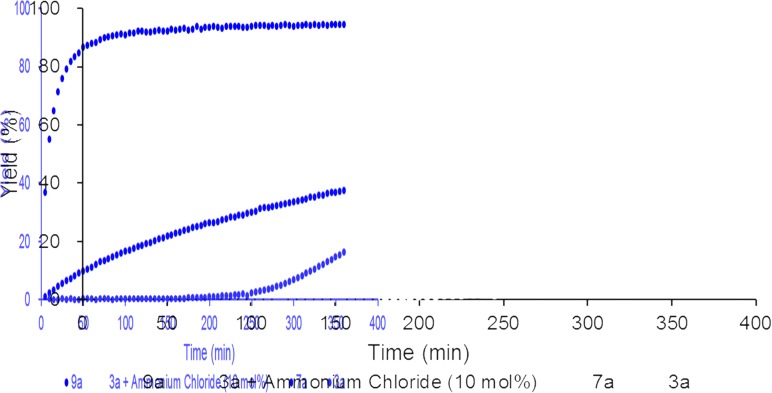
Reaction profile for the hydrodehalogenation catalyzed by Mo_2_ complexes (3 mol%) with MBTCD (1.2 equiv.) at 60 °C.

To reveal the additive effects of ^*n*^Bu_4_NCl to **3a**, we conducted control experiments. We observed the formation of trimethylsilyltoluene and ClSiMe_3_ as the reaction byproducts derived from MBTCD, along with Me_3_SiOAc, which was the reaction product of the acetate ligand and ClSiMe_3_. Because the ligand replacement reaction of an acetate ligand by ClSiMe_3_ was reported by Cotton *et al.*,^14^ we examined the reaction of **3a** with excess ClSiMe_3_ in CD_3_CN (Scheme 1). In the ^1^H NMR spectrum, new resonances assignable to the Mo_2_ complex **6a** appeared within 30 minutes at room temperature, and, after 41 h, all of **3a** was converted to **7a**, which was isolated as a purple powder.^15^ Subsequent heating of a solution of **7a** at 80 °C resulted in the formation of a dimeric molybdenum cluster, [Mo_2_{CH(NAr)_2_}_2_(μ-Cl)_2_]_2_ (**8a**),^16^ which was previously isolated and structurally characterized. The dimeric complex **8a** exhibited low catalytic activity even in the presence of ^*n*^Bu_4_NCl, probably due to the low solubility of **8a** in the reaction media (run 13). In contrast, treatment of complex **3a** with ClSiMe_3_ in the presence of ^*n*^Bu_4_NCl in toluene at 80 °C afforded a dianionic Mo_2_ species, [^*n*^Bu_4_N]_2_[Mo_2_{CH(NAr)_2_}_2_Cl_4_] (**9a**), in which two amidinates and four chloride ligands coordinated to the Mo_2_ core, based on single crystal X-ray diffraction analysis (Fig. 2(a)).^15^,^17^ Notably, no conversion of **9a** to the Cl-bridged dimer **8a** was observed, even upon prolonged heating of **9a** in CH_3_CN. The catalytic activity of **9a** was equal to that of **3a**/^*n*^Bu_4_NCl (runs 10 and 14). By monitoring the reaction progress using ^1^H NMR spectroscopy, we found the induction period for the **3a**/^*n*^Bu_4_NCl catalyst. Complexes **3a** or **7a** were slowly catalyzing the hydrodehalogenation reaction. Interestingly, complex **9a** initiated the catalytic hydrodehalogenation reaction without any induction period (Fig. 3), indicating that the dianionic complex **9a** was the identity of the catalytically active species.

**Scheme 2:**
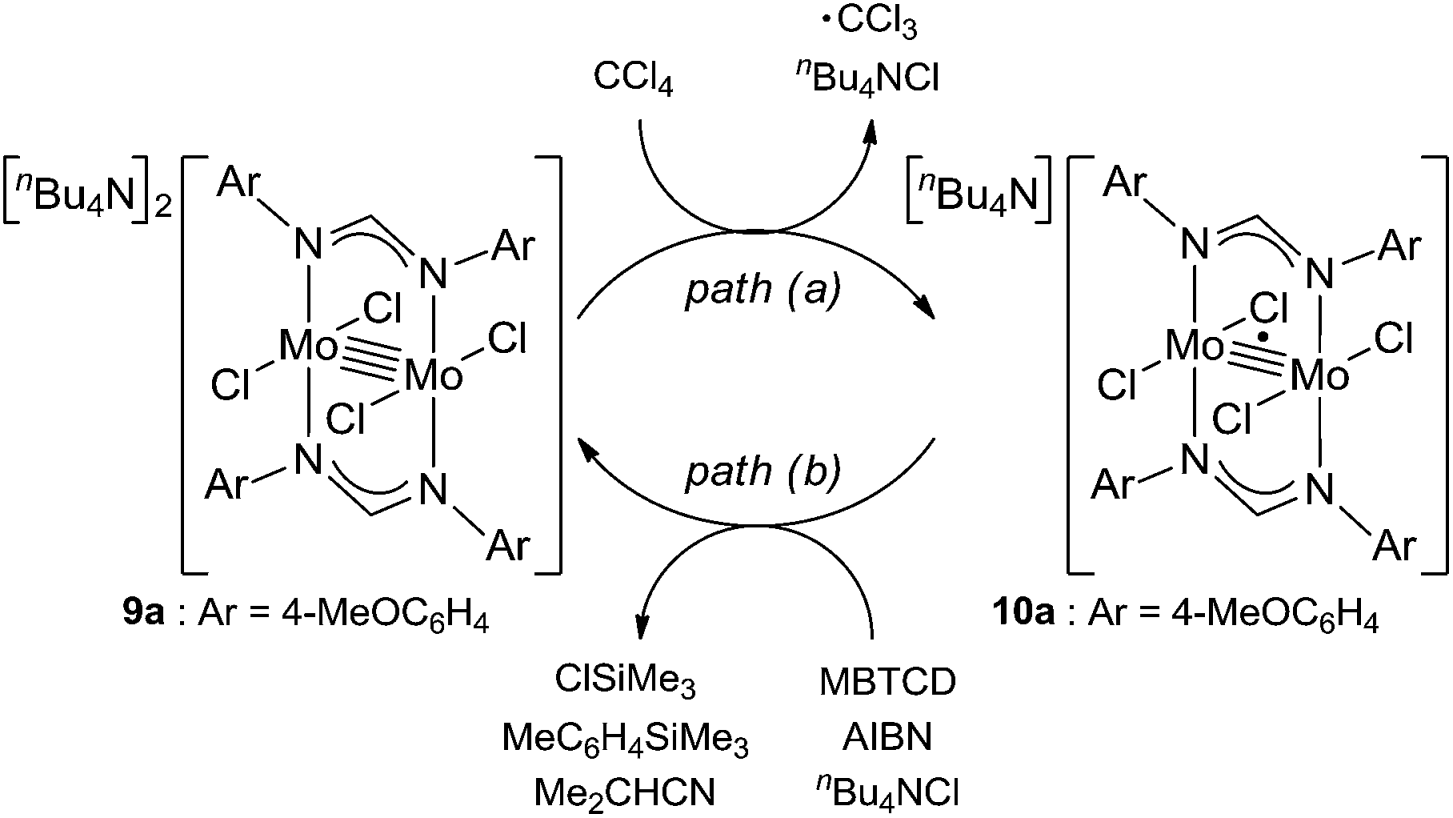
Redox reactions of **9a** and **10a**.

Because of the inactivity of MBTCD toward [Mo_2_]^4+^ complexes, the catalytic reaction of **9a** (**3a**/^*n*^Bu_4_NCl) was assumed to be initiated by the first reductive cleavage of a carbon–halogen bond, which produced a carbon radical and an [Mo_2_]^5+^ species. Thus, we next performed an oxidation reaction of **9a** with CCl_4_ at room temperature, and the oxidized [Mo_2_]^5+^ species, [^*n*^Bu_4_N][Mo_2_{CH(NAr)_2_}_2_Cl_4_] (**10a**), was isolated in 88% yield (Scheme 2, path a). During the reaction, ^*n*^Bu_4_NCl was eliminated, and the ligand system of the Mo_2_ core remained intact. A single crystal X-ray diffraction study of **10a** confirmed the elongation of the Mo–Mo bond by ∼0.04 Å from **9a**, and the distance of the Mo–Mo bond is typical for [Mo_2_]^5+^ species (Fig. 2(b)).8a,^15^,^18^ The formation of [Mo_2_]^5+^ was further confirmed using EPR spectroscopic analysis, in which resonances typical of [Mo_2_]^5+^ species were detected (*g* = 1.955). The high catalytic activity of **9a** was ascribed to the relatively negative *E*_ox_([Mo_2_]^4+/5+^) value: the *E*_ox_ ([Mo_2_]^4+/5+^) value of **9a** was –0.29 V, which shifted to a more negative value compared to that of **7a** (–0.14 V) and **8a** (–0.08 V). The other aspect of the high catalytic activity of **9a** is the stability of the Mo_2_(L)_2_Cl_4_ structure and solubility during the redox processes. In fact, complex **7a** reacted with CCl_4_ to form a [Mo_2_]^6+^ species, [Mo_2_{CH(NAr)_2_}_2_Cl_4_(CH_3_CN)_2_] (**11a**), as poorly soluble dark-red microcrystals that precipitated from the reaction mixture (eqn (1)).^15^
1

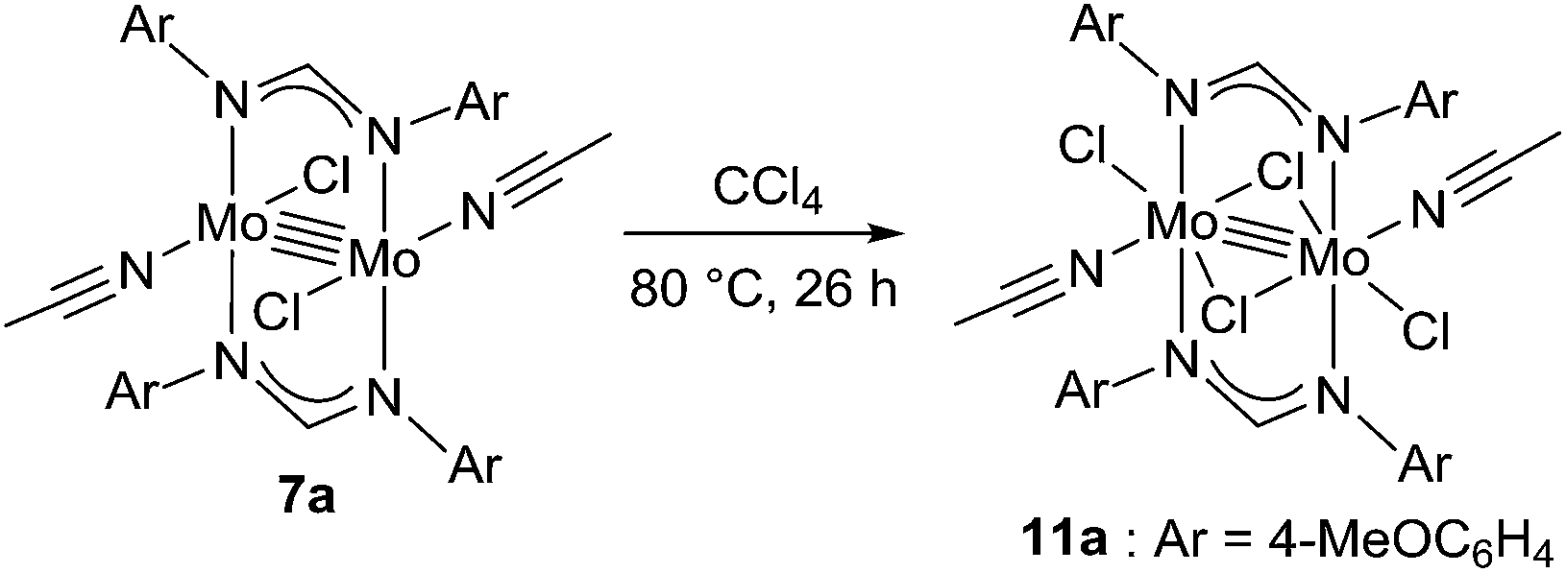




When the oxidized species **10a** was treated with MBTCD in the absence of ^*n*^Bu_4_NCl or without MBTCD in the presence of ^*n*^Bu_4_NCl to reduce **10a**, no reaction was observed, even after heating. On the other hand, in the presence of MBTCD, ^*n*^Bu_4_NCl, and AIBN as a carbon radical source, we observed the formation of **9a** together with ClSiMe_3_, trimethylsilyltoluene, and isobutyronitrile, after heating at 80 °C for 1.5 h (Scheme 2, path b). This indicated that the AIBN-derived carbon radical abstracted one hydrogen atom from MBTCD to generate isobutyronitrile and a radical derivative of MBTCD, which subsequently reduced **10a** to give **9a**, along with ClSiMe_3_ and trimethylsilyltoluene.

**Fig. 4:**
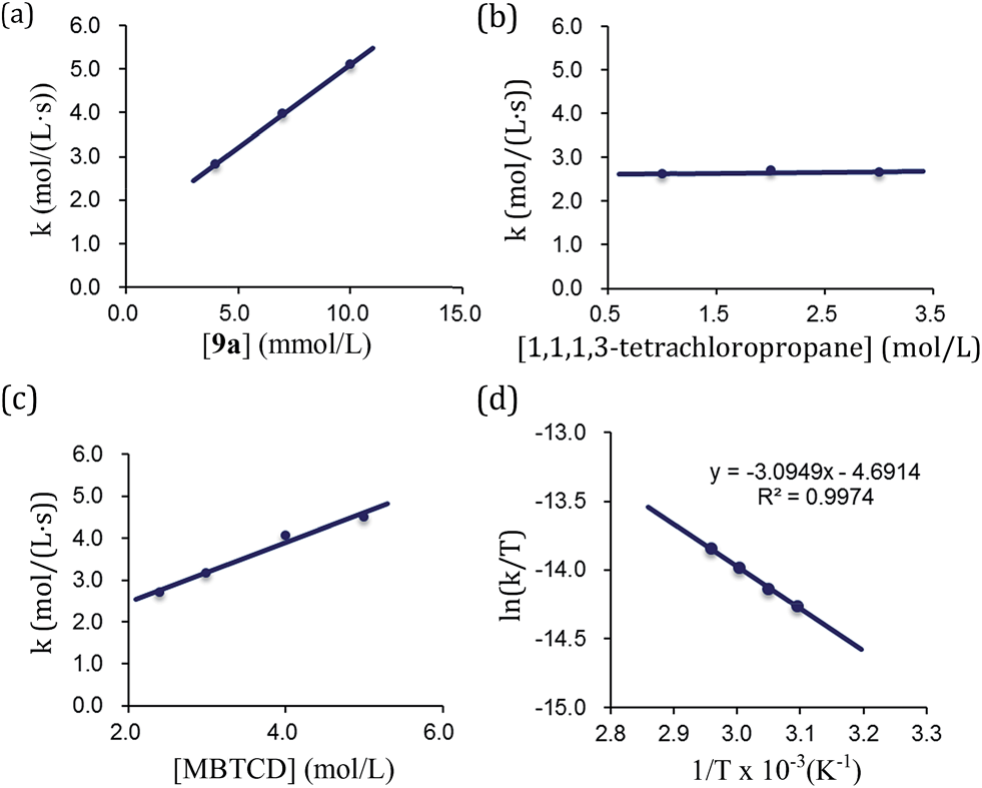
Kinetic analyses of the catalytic hydrodehalogenation reaction catalyzed by **9a**. (a) Rate-dependence on [**9a**]. (b) Rate-dependence on [1,1,1,3-tetrachloropropane]. (c) Rate-dependence on [MBTCD]. (d) Eyring plot in the range of 50–65 °C.

By using the complex **9a** as a catalyst for the hydrodehalogenation of 1,1,1,3-tetrachloropropane, we checked the initial reaction rate dependence for the catalyst, 1,1,1,3-tetrachloropropane, and MBTCD. A first-order rate dependence on the catalyst and MBTCD concentration was observed, whereas the reaction was not dependent on the concentration of 1,1,1,3-tetrachloropropane (Fig. 4(a)–(c)). During the catalytic reaction as described in Table 1, we did not find any byproducts such as radical homo-coupling and disproportionated products, suggesting that the reaction of **9a** and 1,1,1,3-tetrachloropropane is in fast equilibrium with **10a** before reacting with MBTCD. Zero-order in 1,1,1,3-tetrachloropropane might indicate the saturation of the reactive intermediate composed of **10a** and the carbon radical in the coordination sphere, which is consistent with the large negative entropy value (*vide infra*). In addition, MBTCD-*d*_8_ was applied to the catalytic reaction: the KIE value was 1.71, suggesting that the H-abstraction from MBTCD by the organic radical was involved in the rate-determining step. Furthermore, the rate of the catalytic reaction over a temperature ranging from 50 to 65 °C was monitored by ^1^H NMR spectroscopy. Eyring kinetic analyses of the reaction profile afforded the activation parameters of Δ*H*^‡^ = 25.7 ± 0.9 kJ mol^–1^, Δ*S*^‡^ = –56.5 ± 0.7 e.u., and Δ*G*^‡^(298 K) = 96.2 ± 1.8 kJ mol^–1^ (Fig. 4(d)). A large negative Δ*S*^‡^ value indicated an ordered transition state for the H-abstraction step: we presume that the organic radical derived from 1,1,1,3-tetrachloropropane stays in the coordination sphere of **10a** after C–Cl reductive cleavage, while the radical abstracts the H-atom from MBTCD.

**Scheme 3:**
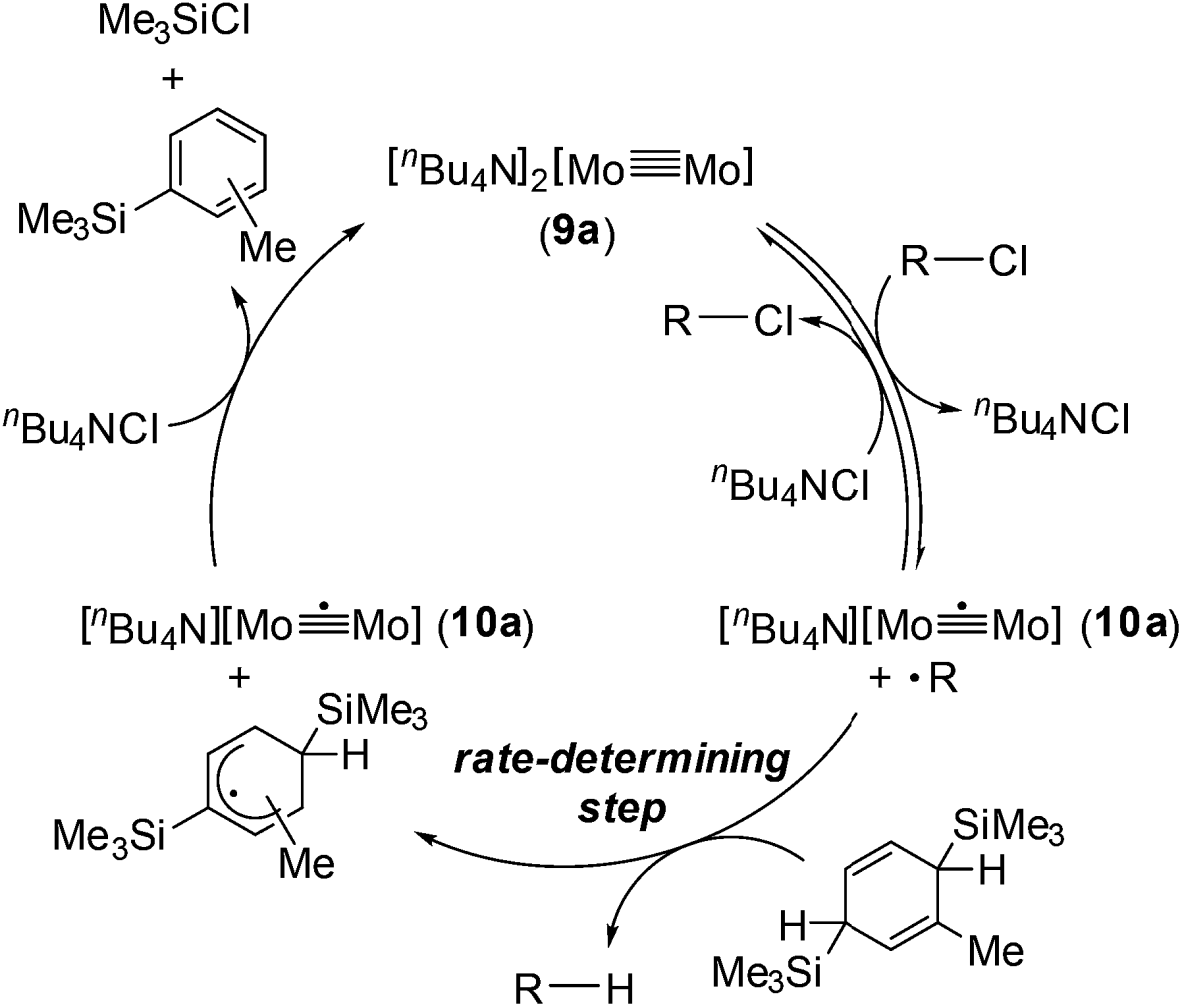
Plausible mechanism for hydrodehalogenation reactions of alkyl halides catalyzed by the Mo_2_ complex **9a**.

Based on the above observations for the kinetic study and redox reactions of the Mo_2_ complexes, we propose a plausible catalytic cycle as shown in Scheme 3. In the initial stage, the dinuclear metal cluster **9a** is in equilibrium with [Mo_2_]^5+^ species **10a** in the presence of alkyl halides, which is often observed for the carbon radical generation by low-valent metal species with alkyl halides.^19^ Because of the zero-order dependence on the substrate concentration and no observation of the radical homo-coupling and disproportionated compounds, this equilibrium is very fast, and the reactive intermediate, **10a** and the carbon radical in the coordination sphere, is generated. Next, the carbon radical abstracts a hydrogen atom from MBTCD, which is a rate-determining step in this catalytic cycle, to form hydrodehalogenated products and a radical derivative of MBTCD. Finally, the [Mo_2_]^5+^ cluster is reduced by the MBTCD-derived radical to regenerate the [Mo_2_]^4+^ species **9a** together with ClSiMe_3_ and trimethylsilyltoluene.

Under optimized conditions for the catalytic hydrodehalogenation reaction using **3a**/^*n*^Bu_4_NCl in CH_3_CN at 80 °C as described in Table 1, the substrate scope was surveyed for haloalkanes having a trichloromethyl or a bromomethyl group, and the results are summarized in Table 2. Tetrachloroalkanes having a longer alkyl chain, ether, and ester groups were effectively dehalogenated to give the corresponding trichloroalkanes in 66%–82% yields (runs 1–3). In addition, α-halocarbonyl ester derivatives and benzyl bromide derivatives were applicable for the hydrodehalogenation reaction in the presence of 2 equiv. of MBTCD to afford the corresponding products in good yields (runs 4–7).

**Table 2:** Substrate scope for hydrodehalogenation reactions catalyzed by **3a**/^*n*^Bu_4_NCl

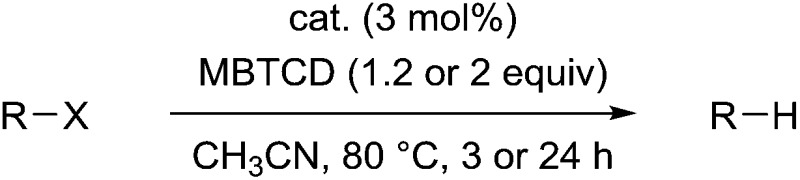
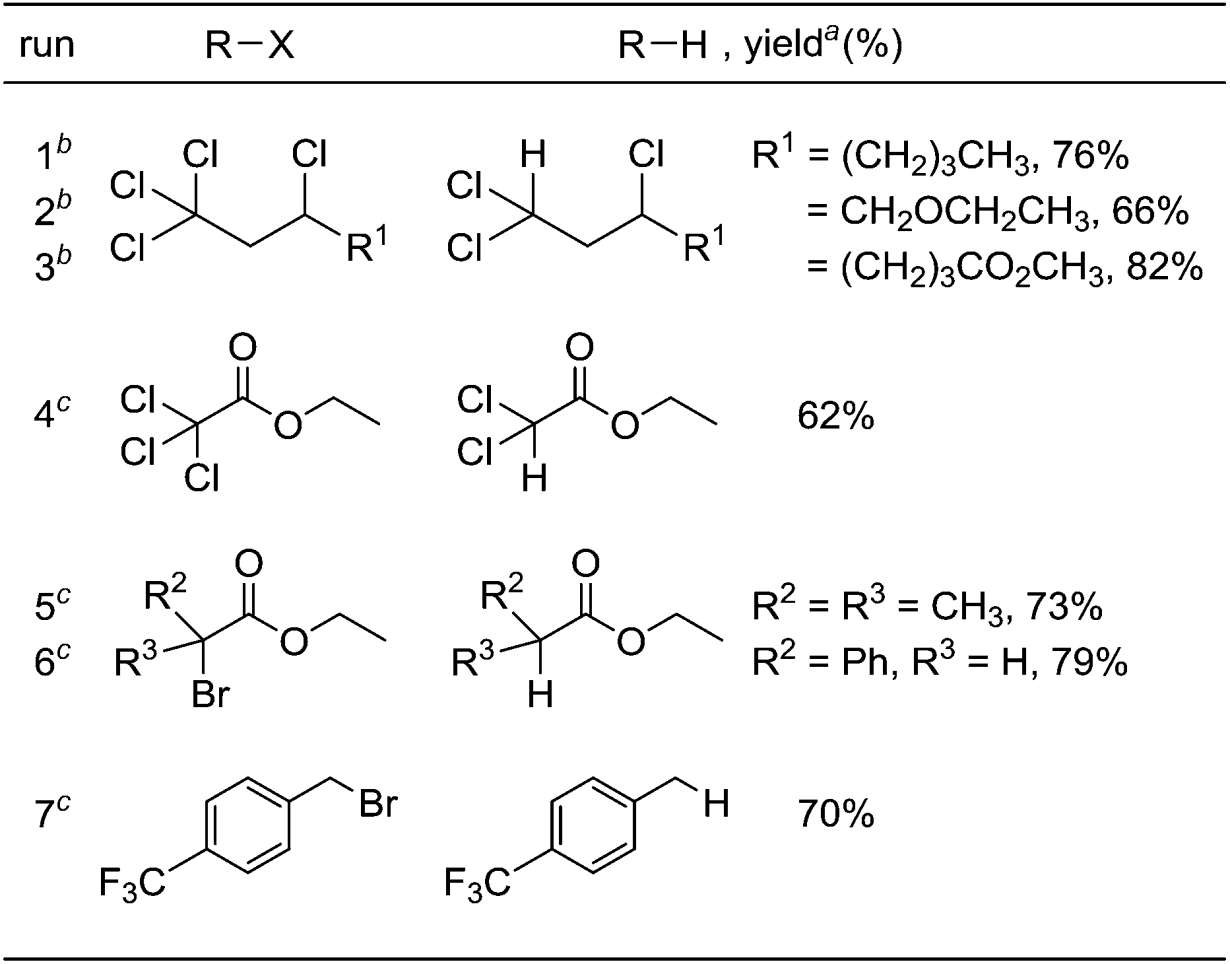

^*a*^Isolated yield for runs 1–3, and NMR yield for runs 4–7.

^*b*^In the presence of 1.2 equiv. of MBTCD for 3 h.

^*c*^In the presence of 2.0 equiv. of MBTCD for 24 h.

## Conclusions

We developed catalytic hydrodehalogenation reactions using paddlewheel Mo_2_ complexes in the presence of MBTCD as an H-atom donor as well as a reductant. *Trans*-Mo_2_(OAc)_2_[CH(NAr)_2_]_2_ (**3a**, Ar = 4-MeOC_6_H_4_) exhibited high catalytic activity for the catalytic reaction in the presence of ^*n*^Bu_4_NCl. Control experiments revealed that the ligand exchange of two acetate ligands of **3a** by ClSiMe_3_ and ^*n*^Bu_4_NCl to form an ionic [^*n*^Bu_4_N]_2_[Mo_2_{CH(NAr)_2_}_2_Cl_4_] (**9a**) were key reaction events to generate the catalytically active species. In addition, kinetic analysis of the catalytic reaction profile, a deuterium-labeling experiment using MBTCD-*d*_8_, and isolation of the oxidized species **10a** clarified the catalytic cycle and the rate-determining step in the catalytic reaction. Further studies of the applications of one electron redox processes by paddlewheel Mo_2_ complexes toward various organic transformations are ongoing in our laboratory.

## Supplementary Material

Supplementary information

Crystal structure data
