# Supplementary material for: Mixed-ligand complexes of paddlewheel dinuclear molybdenum as hydrodehalogenation catalysts for polyhaloalkanes
Source: Chem Sci. 2015 Mar 31;6(6):3434–9. doi: 10.1039/c5sc00721f (PMC5659211; doi:10.1039/c5sc00721f)
Supplement: Supplementary file 1 [file SC-006-C5SC00721F-s001.pdf]

Electronic Supplementary Material (ESI) for Chemical Science.
This journal is © The Royal Society of Chemistry 2015

# Supporting Information

**for**

# Mixed-ligand Complexes of Paddlewheel Dinuclear Molybdenum as Hydrodehalogenation Catalysts for Polyhaloalkanes

Hayato Tsurugi,* Akio Hayakawa, Shun Kando, Yoshitaka Sugino, and Kazushi Mashima*

*Department of Chemistry, Graduate School of Engineering Science, Osaka University, and CREST, JST, Toyonaka, Osaka 560-8531, Japan*

Tel/Fax: +81-6-6850-6245; E-mail: mashima@chem.es.osaka-u.ac.jp

1. **Experimental Section**

   1-1. **General Procedure**

   1-2. **General Procedure for Hydrodehalogenation Reaction of 1,1,1,3-Tetrachloro- propane Catalyzed by $Mo_2$ Clusters (Table 1)**

   1-3. **Generation of $[Mo_2(OAc)\{(ArN)_2CH\}_2Cl(CH_3CN)]$ (6a).**

   1-4. **Preparation of $[Mo_2\{(ArN)_2CH\}_2Cl_2(CH_3CN)_2]$ (7a).**

   1-5. **Preparation of $[Mo_2\{(ArN)_2CH\}_2(\mu\text{-}Cl)_2]_2$ (8a).**

   1-6. **Preparation of $[^{n}Bu_4N]_2[Mo_2\{(ArN)_2CH\}_2Cl_4]$ (9a).**

   1-7. **Preparation of $[^{n}Bu_4N][Mo_2\{(ArN)_2CH\}_2Cl_4]$ (10a).**

   1-8. **Reaction of 7a with $CCl_4$.**

   1-9. **Reduction of 10a by MBTCD and AIBN.**

   1-10. **CV of $Mo_2$ Complexes.**

   1-11. **General Procedure for Hydrodehalogenation Reaction of Haloalkanes Catalyzed by $Mo_2$ Clusters (Table 2).**

   1-12. **Details for X-Ray Crystallographic Analysis.**

2. **Characterization of Hydrodehalogenated Product.**

3. **Kinetic Study for Hydrodehalogenation Reaction**

4. **Molecular Structure of Dinuclear Molybdenum Complexes.**

5. **References**

## 1. Experimental Section

### 1-1. General Procedures.

All manipulations involving air- and moisture-sensitive $Mo_2$ complexes were operated using standard Schlenk or glovebox techniques under argon. MBTCD[S1] and molybdenum complexes ($Mo_2(OAc)_4$ (**1**),[S2] $Mo_2(OAc)_3[(ArN)_2CH]$ (Ar = 2-$MeOC_6H_4$, **2**),[S3] $Mo_2(OAc)_2[(ArN)_2CH]_2$ (Ar = 4-$MeOC_6H_4$, **3a** and **3b**; Ar = 2,6-$Me_2C_6H_4$, **3c**),[S4] $Mo_2(OAc)[(ArN)_2CH]_3$ (Ar = 4-$MeOC_6H_4$, **4**),[S5] and $Mo_2[(ArN)_2CH]_4$ (Ar = 4-$MeOC_6H_4$, **5**)[S6]) were prepared according to the literatures. Anhydrous hexane, toluene, THF, $Et_2O$, acetonitrile, and dichloromethane were purchased from Kanto Chemical, and further purified by passage through activated alumina under positive argon pressure as described by Grubbs *et al.*[S7] Other organic substrates and $^n$$Bu_4NCl$ were purchased, and dried and deoxygenated. Benzene-$d_6$, toluene-$d_8$, $CDCl_3$, $CD_2Cl_2$, $CD_3CN$, and THF-$d_8$ were dried and degassed by $CaH_2$, and stored under argon.

$^1$H NMR (400 MHz) and $^{13}$C NMR (100 MHz) spectra were measured on BRUKER AVANCEIII-400 spectrometer. Assignments for $^1$H and $^{13}$C NMR peaks for some of the complexes were aided by 2D $^1$H-$^1$H COSY, 2D $^1$H-$^1$H NOESY, 2D $^1$H-$^{13}$C HMQC, and 2D $^1$H-$^{13}$C HMBC spectra. UV-vis spectra were recorded on Aglient 8453 spectrometer. Cyclic voltammograms were measured by BAS ALS610D spectrometer using a standard three-electrode configuration with working electrode (glassy carbon), counter electrode (platinum), and reference electrode (silver wire). [$^n$$Bu_4N$][$PF_6$] was used as the electrolyte, and $Cp_2Fe$ was used as the standard. GC-MS measurement was carried out using a DB-1 capillary column (0.25 mm x 30 m) on a Shimadzu GCMS-QP2010Plus. ESI-mass spectrometric data was obtained using BRUKER microTOF-II spectrometer. The elemental analyses were recorded by using Perkin Elmer 2400 at the Faculty of Engineering Science, Osaka University.

**1-2. General Procedure for Hydrodehalogenation Reaction of 1,1,1,3-Tetrachloropropane Catalyzed by $Mo_2$ Clusters (Table 1).**

A solution of 1,1,1,3-tetrachloropropane (0.10 mmol), MBTCD (1.2 equiv to 1,1,1,3-tetrachloropropane), $Mo_2$ cat. (3 mol% to 1,1,1,3-tetrachloropropane), and 1,4-bis(trifluoromethyl)benzene (10 µL) as an internal standard in 0.5 mL of deuterated solvent was prepared in a light shielded J-Young NMR tube. The reaction mixture was heated to 80 ºC. The conversions and yields were calculated by the $^1$H NMR measurement based on the amount of the internal standard. After the certain reaction time, the reaction mixture was quenched by exposing to air. The reaction mixture was concentrated and purified by column chromatography on a silica gel and Kugelrohr distillation. Because of the similar boiling point of 1,1,3-trichloropropane and trimethylsilyltoluene, which was a reaction byproduct derived from MBTCD, both of compounds were isolated as 3:1 mixture (determined by $^1$H NMR).

**1-3. Generation of $[Mo_2(OAc)\{(ArN)_2CH\}_2Cl(CH_3CN)]$ (6a).**

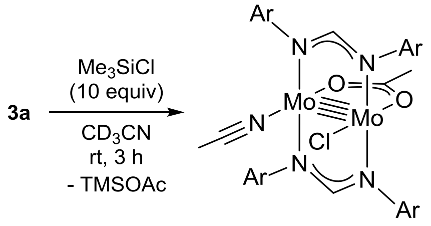


**6a** : Ar = 4-MeOC$_6$H$_4$

$Me_3SiCl$ (10 µL, 79 µmol) was added to a suspension of $Mo_2(OAc)_2[(ArN)_2CH]_2$ (Ar = 4-MeOC$_6$H$_4$) (**3a**, 6.5 mg, 7.9 µmol) in $CD_3CN/CD_2Cl_2$ (0.5 mL, v/v = 1/1). The reaction mixture changed gradually to pale orange suspension. The formation of $[Mo_2(OAc)\{(ArN)_2CH\}_2Cl(CH_3CN)]$ (Ar = 4-MeOC$_6$H$_4$, **6a**) and 1 equiv of $Me_3SiOAc$ were confirmed by the $^1$H NMR spectrum (Figure S1). $^1$H NMR (400 MHz, 30 ºC, $CD_3CN/CD_2Cl_2$ = 1/1): δ 8.85 (s, 2H, NC*H*N), 6.79 (m, 16H, *Ar*), 3.72 (s, 6H, OC*H*$_3$) 3.71 (s, 6H, OC*H*$_3$), 2.47 (s, 3H, O$_2$CC*H*$_3$).

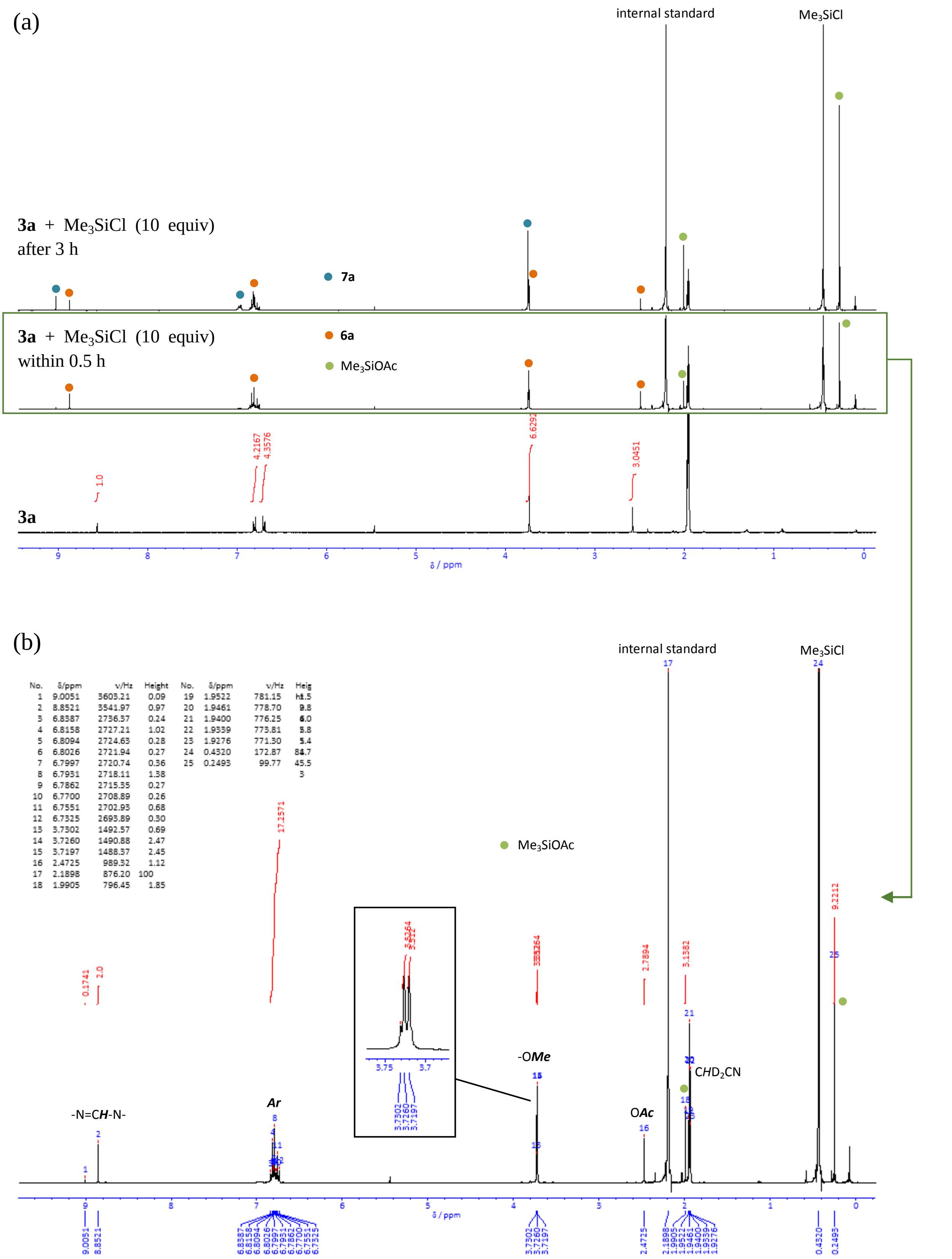


**Figure S1.** $^{1}$H NMR spectral changes for the formation of **6a** from **3a**. (a) $^{1}$H NMR spectra of **3a**, **3a** and $Me_3SiCl$ (10 equiv) in $CD_3CN/CD_2Cl_2$ within 0.5 h, and after 3h. (b) $^{1}$H NMR spectrum of **3a** and $Me_3SiCl$ (10 equiv) in $CD_3CN/CD_2Cl_2$ within 0.5 h.

## 1-4. Preparation of $[Mo_2\{(ArN)_2CH\}_2Cl_2(CH_3CN)_2]$ (7a).

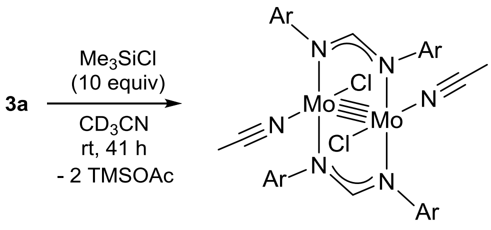


**7a** : Ar = 4-MeOC$_6$H$_4$

To a yellow suspension of $Mo_2(OAc)_2[(ArN)_2CH]_2$ (Ar = 4-MeOC$_6$H$_4$) (0.324 g, 0.395 mmol) in acetonitrile (20 mL), $Me_3SiCl$ (0.500 mL, 3.94 mmol) was added at room temperature, and then the yellow suspension gradually changed to a pale red suspension. The mixture was further stirred at room temperature for 41 h. After the supernatant was decanted, the pale red solid was washed with acetonitrile (3 x 10 mL). All volatiles were removed under vacuum to afford purple powders of **7a** (0.301 g, 0.352 mmol, 89 %), mp 211–215 °C (dec). $^1$H NMR (400 MHz, 30 °C, $CD_3CN/CD_2Cl_2$ = 1/1): δ 8.98 (s, 2H, NCHN), 6.93 (br, 8H, Ar), 6.77 (m, 8H, Ar), 3.73 (s, 12H, $OCH_3$), 1.96 (s, 6H, $NCCH_3$). $^{13}$C NMR (100 MHz, 30 °C, $CD_3CN/CD_2Cl_2$ = 1/1): δ 159.9 (NCHN), 156.9 (Ar), 144.0 (Ar), 123.2 (Ar), 114.8 (Ar), 55.8 ($OCH_3$). UV–vis ($CH_2Cl_2$) $\lambda_{max}$/nm ($\varepsilon$ / M$^{-1}$ cm$^{-1}$): 291 (4.2 x 10$^4$), 526 (1.8 x 10$^3$). Anal. Calcd for $C_{34}H_{36}Cl_2Mo_2N_6O_4$: C, 47.73; H, 4.24; N, 9.82. Found: C, 47.54; H, 3.91; N, 9.72.

## 1-5. Preparation of $[Mo_2\{(ArN)_2CH\}_2(\mu\text{-}Cl)_2]_2$ (8a).[S8)]

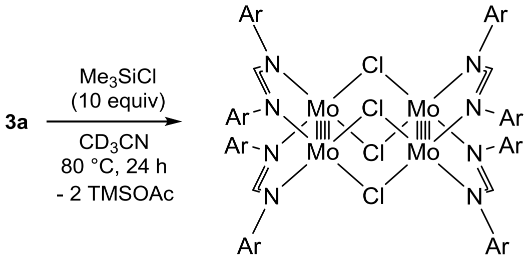


**8a** : Ar = 4-MeOC$_6$H$_4$

A mixture of $Mo_2(OAc)_2[(ArN)_2CH]_2$ (Ar = 4-MeOC$_6$H$_4$) (6.5 mg, 7.9 µmol) and $Me_3SiCl$ (10 µL, 79 µmol) in acetonitrile (0.5 mL) was heated to reflux for 24 h. The yellow suspension gradually changed to a pale orange suspension. After the supernatant was decanted, red purple

crystals were obtained. The $^{1}$H NMR spectrum and the result of X-ray diffraction study were consistent with those reported data by Cotton *et al.*

### 1-6. Preparation of [$^{n}$Bu$_4$N]$_2$[Mo$_2${(ArN)$_2$CH}$_2$Cl$_4$] (9a).

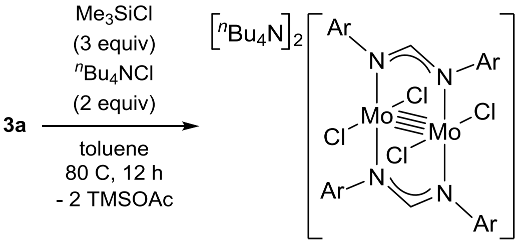


**9a** : Ar = 4-MeOC$_6$H$_4$

To a suspension of Mo$_2$(OAc)$_2$[(ArN)$_2$CH]$_2$ (Ar = 4-MeOC$_6$H$_4$) (0.500 g, 0.609 mmol) and $^{n}$Bu$_4$NCl (0.345 g, 1.24 mmol) in toluene (20 mL), MeSiCl (0.240 mL, 1.89 mmol) was added. The reaction mixture was heated to 80 ºC for 12 h, and then the supernatant was decanted. The orange solid was washed with toluene (6 x 20 mL), THF (3 x 20 mL), and hexane (3 x 10 mL) and dried under vacuum to give orange powders of **9a** (0.737 g, 0.547 mmol, 91 %), mp 198–201 ºC (dec). $^{1}$H NMR (400 MHz, 30 ºC, CD$_3$CN): δ 8.88 (s, 2H, NCHN), 7.03 (d, *J* = 8.8 Hz, 8H, Ar), 6.75 (d, *J* = 8.8 Hz, 8H, Ar), 3.72 (s, 12H, OCH$_3$), 3.10 (br, 16H, NCH$_2$), 1.60 (m, 16H, NCH$_2$C*H$_2$*), 1.35 (dt, *J* = 7.4 Hz, 16H, C*H$_2$*CH$_3$), 0.96 (t, *J* = 7.4 Hz, 24H, CH$_2$C*H$_3$*). $^{13}$C NMR (100 MHz, 30 ºC, CD$_3$CN): δ 158.9 (NCHN), 156.7 (Ar), 145.7 (Ar), 123.8 (Ar), 114.8 (Ar), 59.4 (NCH$_2$), 56.0 (OCH$_3$), 24.4 (NCH$_2$*C*H$_2$), 20.4 (*C*H$_2$CH$_3$), 13.8 (CH$_2$*C*H$_3$). UV–vis (CH$_3$CN) $\lambda_{max}$/nm ($\varepsilon$ / M$^{-1}$ cm$^{-1}$): 279 (3.0 x 10$^4$), 519 (2.2 x 10$^3$). Anal. Calcd for C$_{62}$H$_{102}$Cl$_4$Mo$_2$N$_6$O$_4$: C, 56.02; H, 7.73; N, 6.32. Found: C, 55.71; H, 7.85; N, 6.53.

### 1-7. Preparation of [$^n$Bu$_4$N][Mo$_2${(ArN)$_2$CH}$_2$Cl$_4$] (10a).

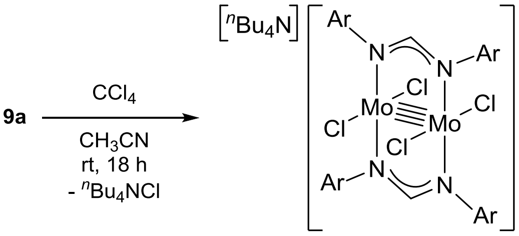


**10a** : Ar = 4-MeOC$_6$H$_4$

$CCl_4$ (0.0150 mL, 0.155 mmol) was added to a solution of [$^n$Bu$_4$N]$_2$[Mo$_2${(ArN)$_2$CH}$_2$Cl$_4$] (Ar = 4-MeOC$_6$H$_4$) (0.200 g, 0.150 mmol) in acetonitrile (20 mL). Upon stirring the reaction mixture, the color of the solution turned dark brown. After stirring at room temperature for 18 h, the solution was concentrated to ca. 3 mL under vacuum. Diethyl ether (30 mL) was added to the reaction mixture, and then brown solids were precipitated. The supernatant was decanted, and the solid was washed with diethyl ether (3 x 10 mL). All the volatiles were removed under vacuum to give brown powders of **10a** (0.145 g, 0.133 mmol, 88 %), mp 225–229 ºC (dec). Some resonances for **10a** were only observed in the $^1$H NMR spectrum. $^1$H NMR (400 MHz, 30 ºC, $CD_3CN$): δ 3.32 (br, 12H, $OCH_3$), 3.08 (br, 8H, $NCH_2$), 1.61 (br, 8H, $NCH_2CH_2$), 1.36 (br, 8H, $CH_2CH_3$), 0.97 (t, $J$ = 7.2 Hz, 24H, $CH_2CH_3$). EPR ($CH_2Cl_2$): $g$ = 1.955. UV–vis ($CH_3CN$) $\lambda_{max}$/nm ($\varepsilon$ / $M^{-1}$ $cm^{-1}$): 237 (3.8 x $10^4$), 272 (3.7 x $10^4$), 476 (1.0 x $10^4$). Anal. Calcd for $C_{46}H_{66}Cl_4Mo_2N_5O_4$: C, 50.84; H, 6.12; N, 6.44. Found: C, 50.80; H, 5.95; N, 6.50.

## 1-8. Reaction of 7a with $CCl_4$.

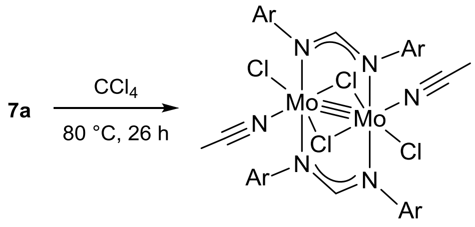


**11a** : Ar = 4-MeOC$_6$H$_4$

$CCl_4$ (5.0 μL, 5.1 μmol) was added to a $CD_3CN$ solution (0.5 mL) of **7a** (4.4 mg, 5.1 μmol) in a light shielded J-Young NMR tube. After heating the reaction mixture at 80 °C for 26 h, sparingly soluble dark-red crystals (**11a**) were formed. X-ray diffraction study of the crystal revealed the formation of $[Mo_2]^{6+}$ species with two amidinate and two chloride ligands.

## 1-9. Reduction of 10a by MBTCD and AIBN.

A solution of $[^nBu_4N][Mo_2\{(ArN)_2CH\}_2Cl_4]$ (**10a**, Ar = 4-MeOC$_6$H$_4$) (6.6 mg, 6.0 μmol), $^nBu_4NCl$ (8.4 mg, 30 μmol), MBTCD (14.5 mg, 60 μmol) and AIBN (5.0 mg, 30 μmol) in 0.5 mL of $CD_3CN$ was prepared in a J-Young NMR tube. The reaction mixture was heated to 80 ºC for 1.5 h. Formation of $[^nBu_4N]_2[Mo_2\{(ArN)_2CH\}_2Cl_4]$ (**9a**, Ar = 4-MeOC$_6$H$_4$) was observed in the $^1$H NMR spectrum.

(a)

| No. | δ/ppm | ν/Hz | Height | No. | δ/ppm | ν/Hz | Height |
|---|---|---|---|---|---|---|---|
| 1 | 7.9161 | 3167.46 | 47.41 | 15 | 1.9624 | 785.22 | 3.58 |
| 2 | 5.6040 | 2242.34 | 1.74 | 16 | 1.9561 | 782.71 | 1.86 |
| 3 | 5.5833 | 2234.06 | 2.50 | 17 | 1.7057 | 682.51 | 77.42 |
| 4 | 5.4776 | 2191.77 | 3.58 | 18 | 1.6664 | 666.77 | 19.57 |
| 5 | 5.4522 | 2181.60 | 1.95 | 19 | 1.6454 | 658.36 | 6.58 |
| 6 | 5.2014 | 2081.22 | 3.22 | 20 | 1.4175 | 567.20 | 3.21 |
| 7 | 3.1855 | 1274.61 | 4.98 | 21 | 1.3997 | 560.04 | 5.64 |
| 8 | 3.1653 | 1266.51 | 4.93 | 22 | 1.3818 | 552.89 | 5.56 |
| 9 | 3.1444 | 1258.17 | 5.09 | 23 | 1.3639 | 545.74 | 3.05 |
| 10 | 2.1644 | 866.03 | 7.48 | 24 | 1.0160 | 406.51 | 10.88 |
| 11 | 2.1600 | 864.28 | 6.61 | 25 | 0.9984 | 399.49 | 21.55 |
| 12 | 1.9806 | 792.50 | 2.18 | 26 | 0.9800 | 392.15 | 9.32 |
| 13 | 1.9745 | 790.05 | 3.84 | 27 | 0.0795 | 31.82 | 100 |
| 14 | 1.9685 | 787.67 | 5.17 | 28 | 0.0160 | 6.41 | 98.27 |

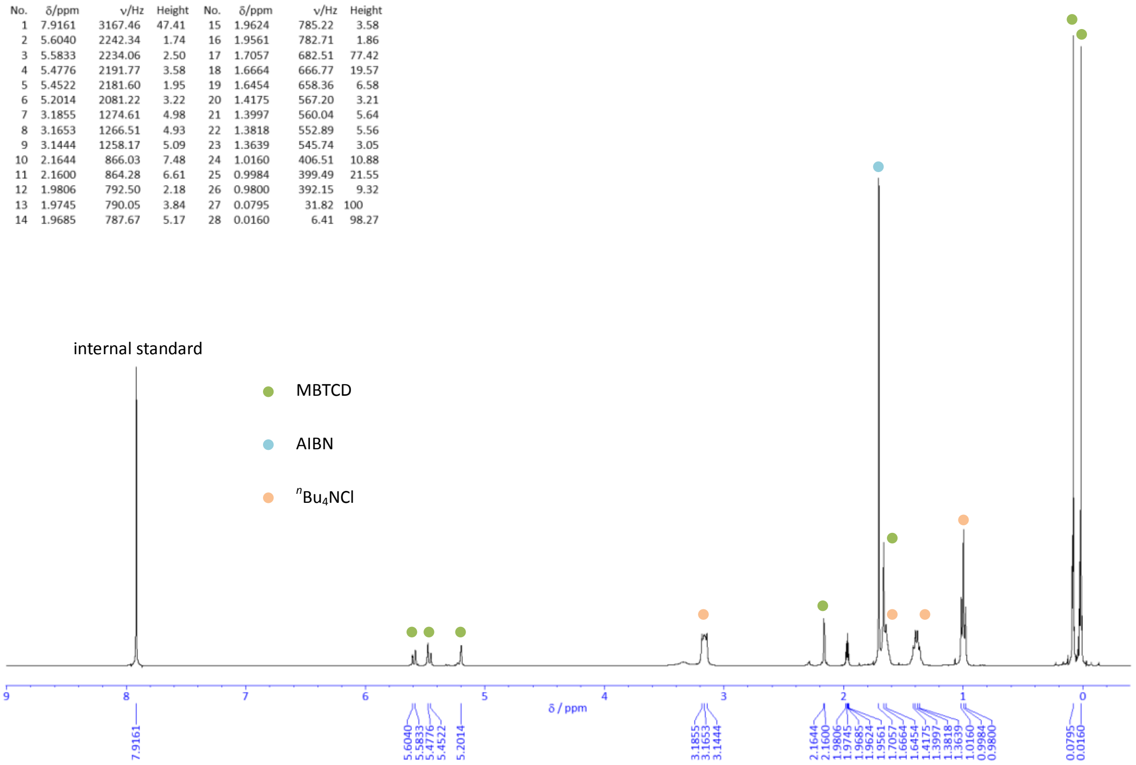


(b)

| No. | δ/ppm | ν/Hz | Height | No. | δ/ppm | ν/Hz | Height | No. | δ/ppm | ν/Hz | Height | No. | δ/ppm | ν/Hz | Height |
|---|---|---|---|---|---|---|---|---|---|---|---|---|---|---|---|
| 1 | 8.8801 | 3553.20 | 0.71 | 16 | 5.1742 | 2070.36 | 2.74 | 31 | 1.9522 | 781.15 | 1.94 | 46 | 1.3664 | 546.74 | 9.15 |
| 2 | 7.8883 | 3156.36 | 47.22 | 17 | 5.1703 | 2068.79 | 2.75 | 32 | 1.9461 | 778.70 | 3.53 | 47 | 1.3479 | 539.34 | 9.30 |
| 3 | 7.0458 | 2819.25 | 0.90 | 18 | 5.1669 | 2067.41 | 1.55 | 33 | 1.9400 | 776.25 | 4.87 | 48 | 1.3296 | 532.00 | 5.28 |
| 4 | 7.0236 | 2810.34 | 1.02 | 19 | 3.7161 | 1486.93 | 5.74 | 34 | 1.9337 | 773.74 | 3.32 | 49 | 1.3114 | 524.72 | 1.41 |
| 5 | 6.7562 | 2703.36 | 1.39 | 20 | 3.1540 | 1262.00 | 8.58 | 35 | 1.9275 | 771.23 | 1.65 | 50 | 1.2695 | 507.97 | 6.68 |
| 6 | 6.7338 | 2694.39 | 1.16 | 21 | 3.1406 | 1256.67 | 4.42 | 36 | 1.6897 | 676.12 | 2.09 | 51 | 1.2519 | 500.94 | 6.49 |
| 7 | 5.5803 | 2232.86 | 1.41 | 22 | 3.1328 | 1253.53 | 5.95 | 37 | 1.6772 | 671.10 | 45.84 | 52 | 0.9846 | 393.97 | 19.76 |
| 8 | 5.5760 | 2231.11 | 1.49 | 23 | 3.1112 | 1244.87 | 8.84 | 38 | 1.6385 | 655.60 | 18.49 | 53 | 0.9664 | 386.69 | 38.83 |
| 9 | 5.5556 | 2222.95 | 2.20 | 24 | 2.7811 | 1112.80 | 0.39 | 39 | 1.6175 | 647.19 | 6.85 | 54 | 0.9479 | 379.29 | 16.18 |
| 10 | 5.5517 | 2221.38 | 2.07 | 25 | 2.7635 | 1105.77 | 0.53 | 40 | 1.5982 | 639.48 | 4.55 | 55 | 0.4229 | 169.23 | 12.05 |
| 11 | 5.4499 | 2180.66 | 2.92 | 26 | 2.7460 | 1098.74 | 0.41 | 41 | 1.5778 | 631.32 | 2.19 | 56 | 0.2346 | 93.87 | 13.17 |
| 12 | 5.4461 | 2179.16 | 1.71 | 27 | 2.4361 | 974.77 | 0.60 | 42 | 1.5497 | 620.09 | 1.35 | 57 | 0.0515 | 20.59 | 100 |
| 13 | 5.4287 | 2172.19 | 1.14 | 28 | 2.3210 | 928.71 | 2.08 | 43 | 1.4724 | 589.16 | 24.70 | 58 | -0.0119 | -4.76 | 99.40 |
| 14 | 5.4248 | 2170.62 | 1.73 | 29 | 2.1363 | 854.81 | 6.71 | 44 | 1.4036 | 561.61 | 1.49 | | | | |
| 15 | 5.1777 | 2071.74 | 1.79 | 30 | 2.1324 | 853.24 | 5.12 | 45 | 1.3851 | 554.21 | 5.41 | | | | |

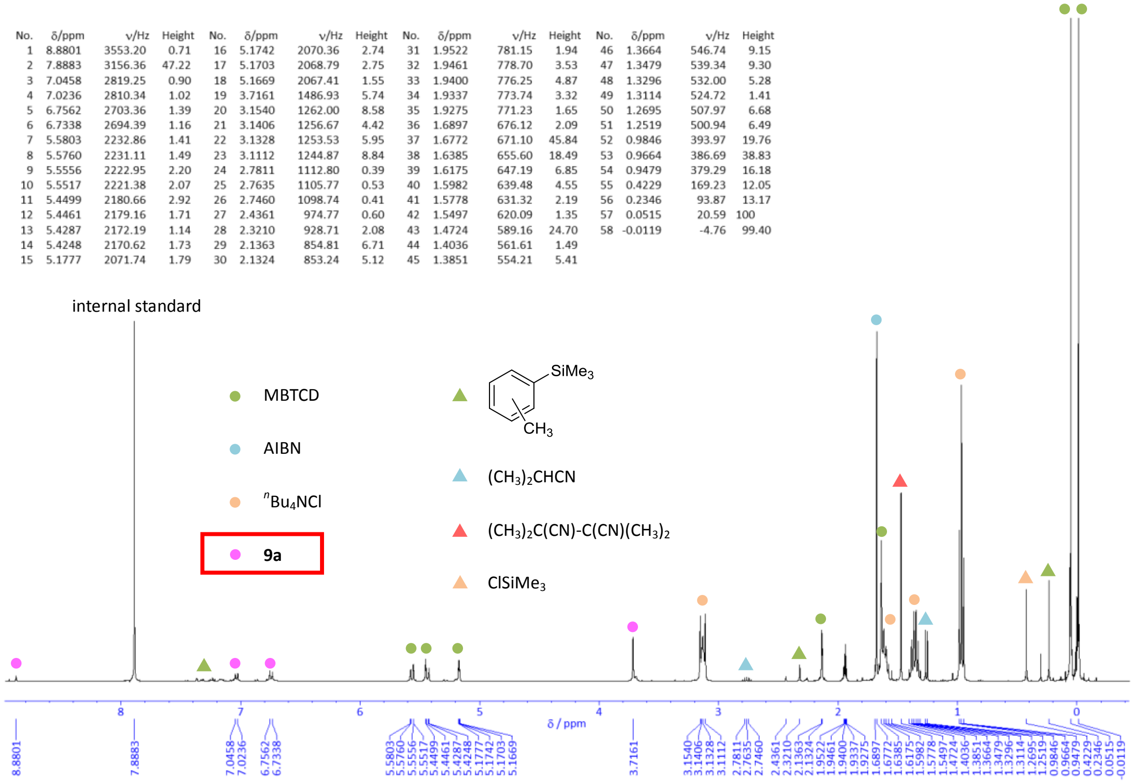


**Figure S2.** $^1$H NMR spectral changes for the formation of **9a** from **10a**. (a) after mixing **10a**, MBTCD, AIBN, and $^n$Bu$_4$NCl. (b) after hearing for 1.5 h at 80 °C.

## 1-10. CV of $Mo_2$ Complexes.

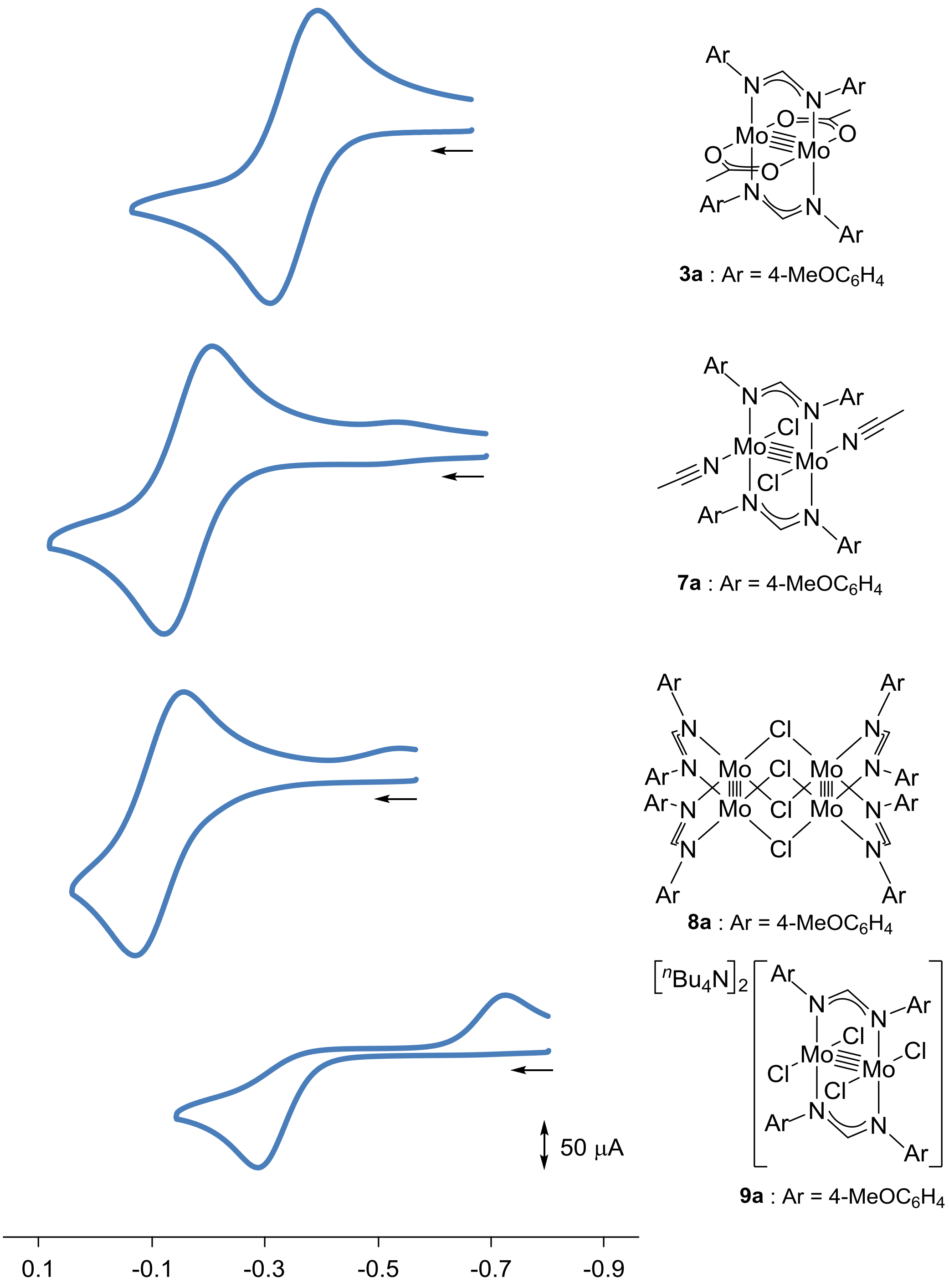


**Figure S3.** CVs for $Mo_2$ complexes **3a**, **7a**, **8a**, and **9a** in $CH_2Cl_2/CH_3CN$ (v/v = 1/1) containing $[^nBu_4N][PF_6]$ as the supporting electrolyte.

**1-11. General Procedure for Hydrodehalogenation Reaction of Haloalkanes Catalyzed by $Mo_2$ Clusters (Table 2).**

A solution of halogenated substrates (1.0 mmol), MBTCD (1.2 equiv), $Mo_2$ catalyst (3 mol%), $^n$$Bu_4NCl$ (10 mol%) in 5 mL of acetonitrile was prepared in a Schlenk tube covered with Al-foil. The reaction mixture was heated to 80 ºC. The reaction mixture was quenched by exposing to air after the reaction time, and all the volatiles were removed by evacuation. Excess $CF_3COOH$ (12 equiv to the substrate) and 3 mL of $CHCl_3$ were added and heated at 60 ºC for 12 h to decompose trimethylsilyltoluene. The reaction mixture was concentrated and purified by Kugelrohr distillation.

**1-12. Details for X-Ray Crystallographic Analysis.**

All crystals were handled similarly. The crystals were mounted on the CryoLoop (Hampton Research Corp.) with a layer of light mineral oil and placed in a nitrogen stream at 113(1) K. Measurements were made on Rigaku AFC7R/Mercury CCD detector with graphite-monochromated Mo–K$\alpha$ (0.71075 Å) radiation. Crystal data and structure refinement parameters were listed in Table S5.

The structures of complexes **7a**, **9a**, **10a**, and **11a** were solved by direct methods (SIR-92).[S9] The structures were refined on $F^2$ by full-matrix least-squares method, using SHELXL-97.[S10] Non-hydrogen atoms were anisotropically refined, except for two carbon atoms of the $^n$$Bu_4N$ cation in **9a**, which were refined in an isotropic manner due to the disorder of those atoms. H-atoms were included in the refinement on calculated positions riding on their carrier atoms. The function minimized was $[\Sigma w(Fo^2 - Fc^2)^2]$ ($w = 1 / [\sigma^2 (Fo^2) + (aP)^2 + bP]$), where $P = (\mathrm{Max}(Fo^2,0) + 2Fc^2) / 3$ with $\sigma^2(Fo^2)$ from counting statistics. The function $R1$ and $wR2$ were

$(\Sigma||Fo| - |Fc||) / \Sigma|Fo|$ and $[\Sigma w(Fo^2 - Fc^2)^2 / \Sigma(wFo^4)]^{1/2}$, respectively. The ORTEP-3 program was used to draw the molecule.[S11]

**2. Characterization of Hydrodehalogenated Product.**

1,1,3-Trichloropropane[S12] (Table 1): $^1$H NMR (400 MHz, 30 ºC, $CDCl_3$): δ 5.96 (t, *J* = 6.3 Hz, 1H, $Cl_2CH$), 3.72 (t, *J* = 6.3 Hz, 2H, $ClCH_2$), 2.63 (q, *J* = 6.3 Hz, 2H, $CH_2$). $^{13}$C NMR (100 MHz, 30 ºC, $CDCl_3$): δ 70.2, 45.6, 40.4.

1,1,3-Trichloroheptane[S13] (run 1 in Table 2): $^1$H NMR (400 MHz, 30 ºC, $CDCl_3$): δ 5.99 (m, 1H, $CHCl_2$), 4.08 (dt, *J* = 13.5 Hz and 6.6 Hz, 1H, CHCl), 2.53 (m, 2H, $CHCl_2CH_2$), 1.77 (dt, *J* = 13.5 Hz and 6.6 Hz, 2H, $CHClCH_2$), 1.58–1.29(m, 4H, $(CH_2)_2CH_3$), 0.93 (t, *J* = 7.2 Hz, 3H, $CH_3$). $^{13}$C NMR (100 MHz, 30 ºC, $CDCl_3$): δ 71.0, 59.6, 51.9, 38.0, 28.4, 22.3, 14.0. HRMS (EI+) m/z calcd. for $C_7H_{13}Cl_3$ 202.0082, found 202.0093.

1,1,3-Trichloro-4-ethoxybutane (run 2 in Table 2): $^1$H NMR (400 MHz, 30 ºC, $CDCl_3$): δ 6.00 (dd, *J* = 9.8 Hz and 3.5 Hz, 1H, $CHCl_2$), 4.20 (m, 1H, CHCl), 3.70–3.53 (m, 4H, $CH_2OCH_2$), 2.73 (m, 1H, $CHCl_2CHH$), 2.54 (m, 1H, $CHCl_2CHH$), 1.22 (t, *J* = 7.0 Hz, 3H, $CH_3$). $^{13}$C NMR (100 MHz, 30 ºC, $CDCl_3$): δ 73.9, 70.7, 67.2, 56.2, 48.8, 15.2. HRMS (EI+) m/z calcd. for $C_6H_{11}O_1Cl_3$ 203.9875, found 203.9876.

Methyl 5,7,7-trichloroheptanoate (run 3 in Table 2): $^1$H NMR (400 MHz, 30 ºC, $CDCl_3$): δ 5.97 (dd, *J* = 7.1 Hz and 6.0 Hz, 1H, $CHCl_2$), 4.08 (m, 1H, CHCl), 3.68 (s, 3H, $CO_2CH_3$) 2.53 (dd, *J* = 7.2 Hz and 6.1 Hz, 2H, $CHCl_2CH_2$), 2.36 (dd, *J* = 8.5 Hz and 4.9 Hz, 2H, $CHClCH_2$), 1.97–1.76

(m, 4H, (C$H_2$)$_2$CO). $^{13}$C NMR (100 MH z, 30 ºC, $CDCl_3$): δ 173.5, 70.7, 58.9, 51.8, 51.7, 37.4, 33.3, 21.7. HRMS (FAB+) m/z calcd. for $C_8H_{14}O_2Cl_3$ 247.0059, found 247.0060.

The $^1$H NMR spectra of hydrodehalogenated products in runs 4-7 (Table 2) were superimposed to the corresponding commercially available compounds.

Ethyl dichloroacetate (run 4 in Table 2): $^1$H NMR (400 MHz, 30 ºC, $CD_3CN$): δ 6.22 (s, 1H, $CHCl_2$), 4.30 (q, $J$ = 7.2 Hz, 3H, OC$H_2$CH$_3$), 1.30 (t, $J$ = 7.2 Hz, 3H, OCH$_2$C$H_3$).

Ethyl isobutyrate (run 5 in Table 2): $^1$H NMR (400 MHz, 30 ºC, $CD_3CN$): δ 4.07 (q, $J$ = 7.2 Hz, 3H, OC$H_2$CH$_3$), 2.50 (sep, $J$ = 6.9 Hz, 1H, C$H$(CH$_3$)$_2$), 1.20 (t, $J$ = 7.2 Hz, 3H, OCH$_2$C$H_3$), 1.12 (d, $J$ = 6.9 Hz, CH(C$H_3$)$_2$).

Ethyl phenylaetate (run 6 in Table 2): $^1$H NMR (400 MHz, 30 ºC, $CD_3CN$): δ 7.2-7.4 (m, 5H, Ph), 4.12 (q, $J$ = 7.1 Hz, 3H, OC$H_2$CH$_3$), 3.62 (s, 2H, C$H_2$Ph), 1.22 (t, $J$ = 7.1 Hz, 3H, OCH$_2$C$H_3$).

4-Trifluorotoluene (run 7 in Table 2): $^1$H NMR (400 MHz, 30 ºC, $CD_3CN$): δ 7.56 (d, $J$ = 7.4 Hz, 2H, Ar), 7.36 (d, $J$ = 7.4 Hz, 2H, Ar), 4.61 (s, 3H, $CH_3$).

| | | | | | | | |
|---|---|---|---|---|---|---|---|
| 1 | 7.2600 | 2904.94 | 9.59 | 9 | 2.6390 | 1055.95 | 26.88 |
| 2 | 5.9791 | 2392.41 | 8.08 | 10 | 2.6235 | 1049.73 | 26.10 |
| 3 | 5.9632 | 2386.07 | 16.42 | 11 | 2.6080 | 1043.52 | 9.06 |
| 4 | 5.9476 | 2379.80 | 8.24 | 12 | 2.3640 | 945.90 | 22.47 |
| 5 | 3.7335 | 1493.88 | 17.25 | 13 | 0.3288 | 131.57 | 15.91 |
| 6 | 3.7181 | 1487.73 | 33.16 | 14 | 0.2642 | 105.72 | 100 |
| 7 | 3.7028 | 1481.59 | 18.19 | 15 | 0.2564 | 102.58 | 14.91 |
| 8 | 2.6545 | 1062.16 | 9.73 | | | | |

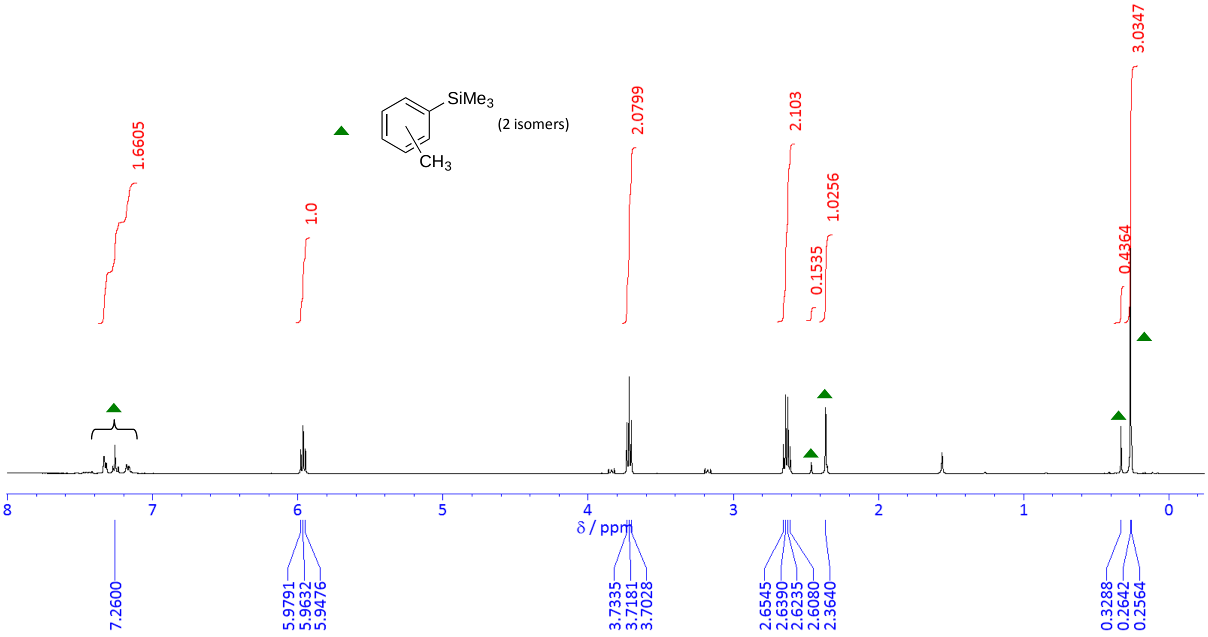


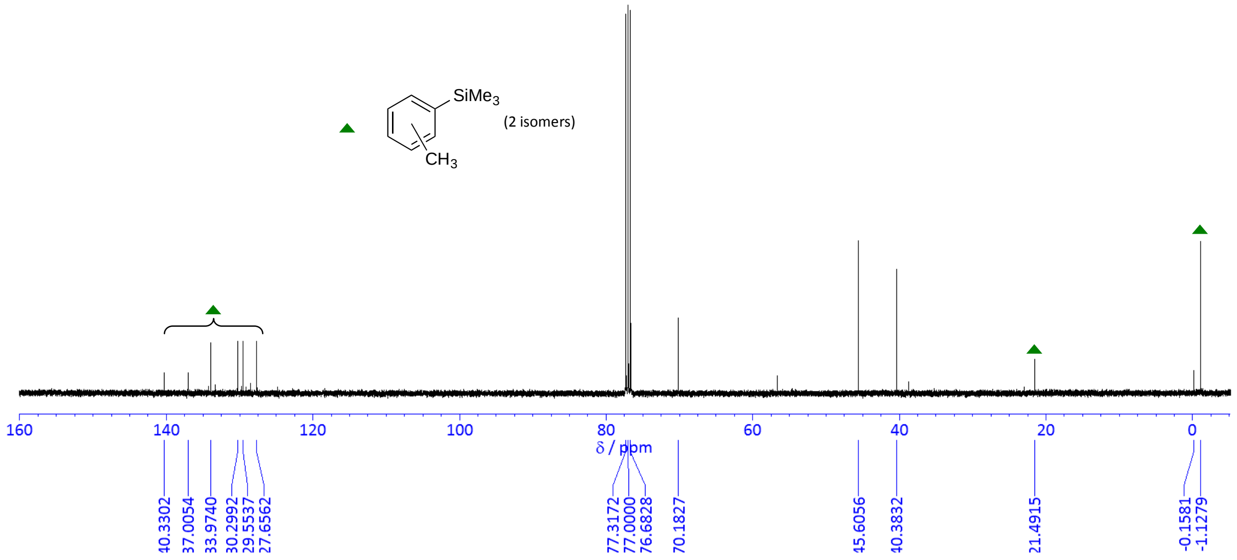


**Figure S4.** $^{1}H$ and $^{13}C$ NMR spectra of 1,1,3-trichloropropane (trimethylsilyltoluene (ca. 25%) was contaminated due to the similar boiling point to 1,1,3-trichloropropane):

| No. | δ/ppm | ν/Hz | Height | No. | δ/ppm | ν/Hz | Height |
|---|---|---|---|---|---|---|---|
| 1 | 7.2600 | 2904.94 | 35.07 | 16 | 1.5263 | 610.73 | 14.16 |
| 2 | 6.0026 | 2401.82 | 16.96 | 17 | 1.5113 | 604.71 | 25.41 |
| 3 | 5.9844 | 2394.54 | 18.40 | 18 | 1.4907 | 596.49 | 12.42 |
| 4 | 5.9698 | 2388.70 | 17.13 | 19 | 1.4489 | 579.74 | 12.38 |
| 5 | 4.0938 | 1638.06 | 11.41 | 20 | 1.4302 | 572.27 | 13.47 |
| 6 | 4.0770 | 1631.35 | 16.02 | 21 | 1.4098 | 564.11 | 12.46 |
| 7 | 4.0607 | 1624.82 | 11.89 | 22 | 1.3902 | 556.27 | 16.40 |
| 8 | 2.5468 | 1019.05 | 28.87 | 23 | 1.3752 | 550.25 | 23.64 |
| 9 | 2.5316 | 1012.97 | 38.88 | 24 | 1.3575 | 543.16 | 26.79 |
| 10 | 2.5234 | 1009.70 | 22.26 | 25 | 1.3391 | 535.82 | 19.88 |
| 11 | 2.5167 | 1007.01 | 26.43 | 26 | 1.3193 | 527.91 | 10.18 |
| 12 | 1.7998 | 720.15 | 14.86 | 27 | 0.9484 | 379.46 | 55.43 |
| 13 | 1.7818 | 712.94 | 39.28 | 28 | 0.9305 | 372.31 | 100 |
| 14 | 1.7637 | 705.72 | 42.13 | 29 | 0.9124 | 365.10 | 45.11 |
| 15 | 1.7457 | 698.51 | 19.69 | | | | |

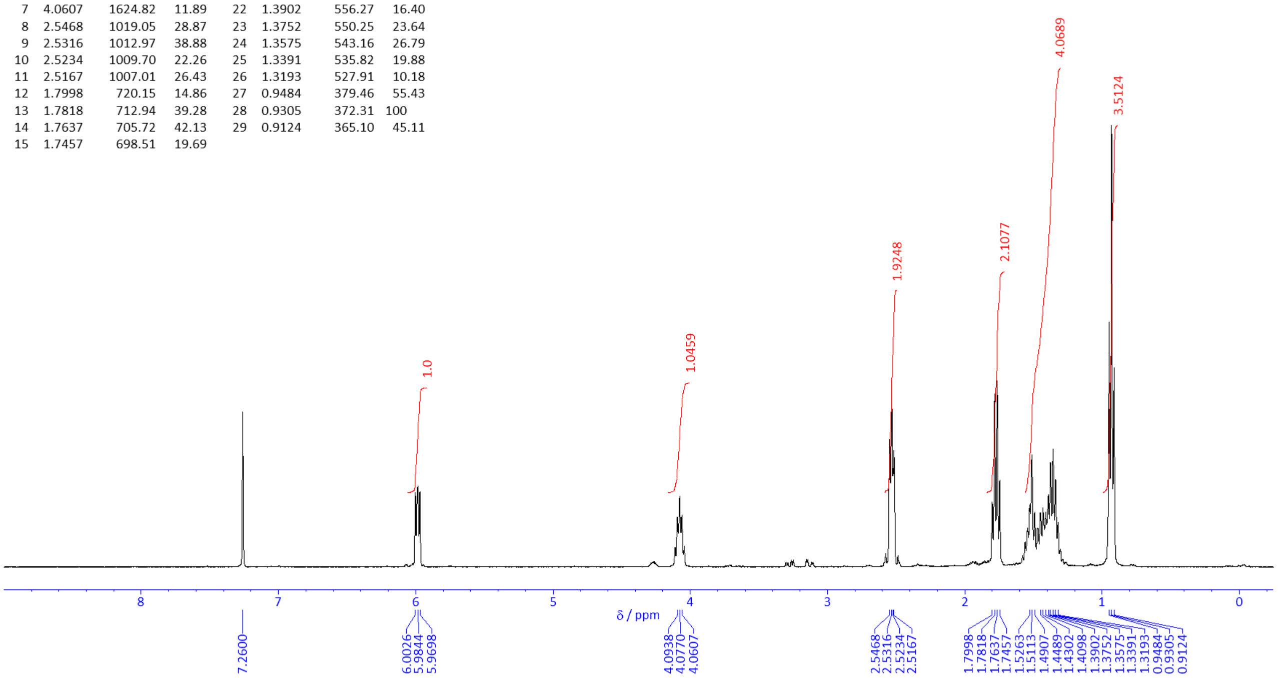


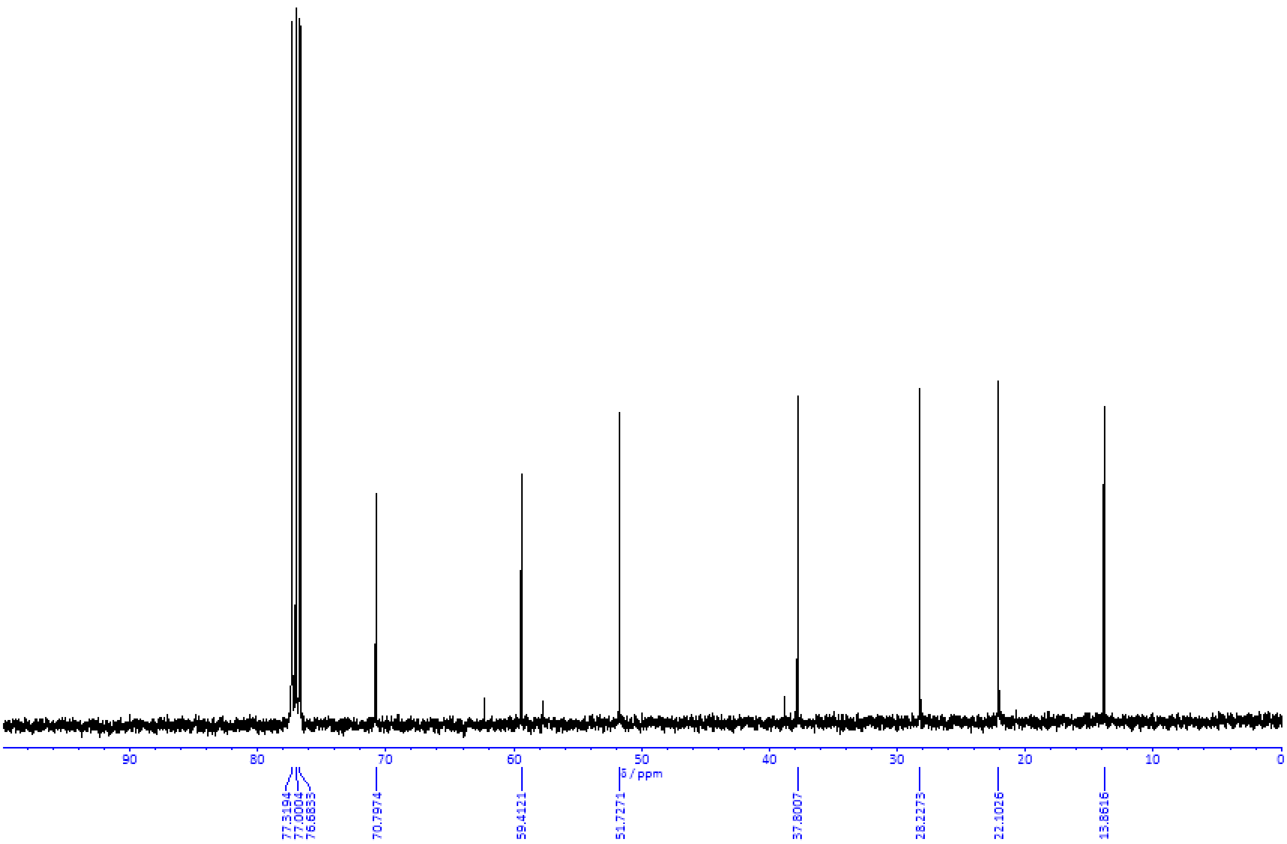


**Figure S5.** $^{1}$H and $^{13}$C NMR spectra of 1,1,3-trichloroheptane:

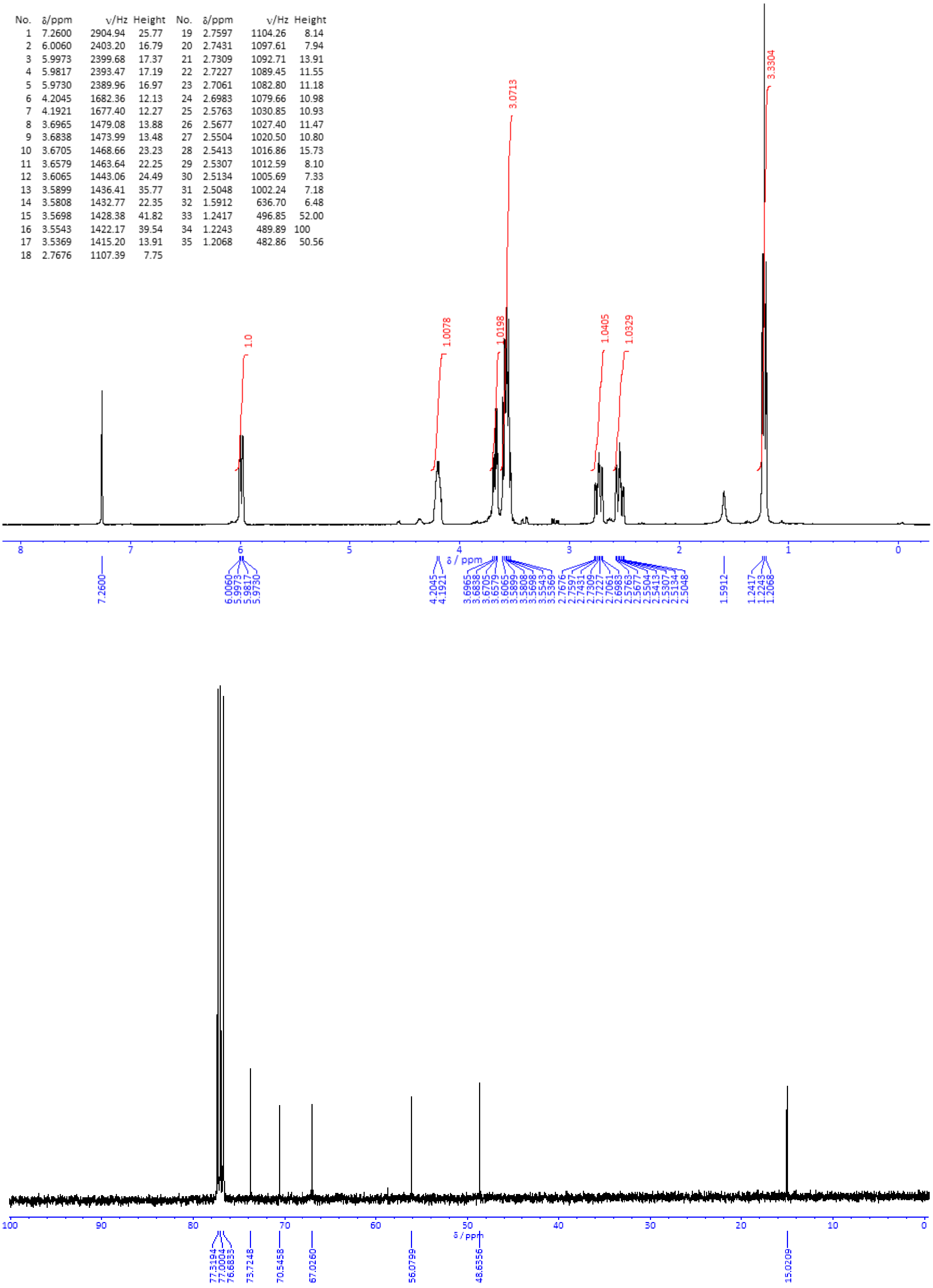


**Figure S6.** $^{1}$H and $^{13}$C NMR spectra of 1,1,3-trichloro-4-ethoxybutane:

| No. | δ/ppm | ν/Hz | Height | No. | δ/ppm | ν/Hz | Height |
|---|---|---|---|---|---|---|---|
| 1 | 7.2600 | 2904.94 | 15.42 | 9 | 2.5216 | 1008.95 | 15.75 |
| 2 | 5.9922 | 2397.68 | 7.85 | 10 | 2.3833 | 953.61 | 12.41 |
| 3 | 5.9747 | 2390.65 | 10.91 | 11 | 2.3677 | 947.40 | 21.06 |
| 4 | 5.9595 | 2384.56 | 7.98 | 12 | 1.8450 | 738.22 | 7.32 |
| 5 | 4.0827 | 1633.61 | 6.21 | 13 | 1.8341 | 733.89 | 9.64 |
| 6 | 3.6865 | 1475.06 | 100 | 14 | 1.8068 | 722.97 | 19.21 |
| 7 | 2.5548 | 1022.25 | 16.92 | 15 | 1.7915 | 716.83 | 15.13 |
| 8 | 2.5393 | 1016.04 | 22.55 | 16 | 1.7673 | 707.16 | 6.58 |

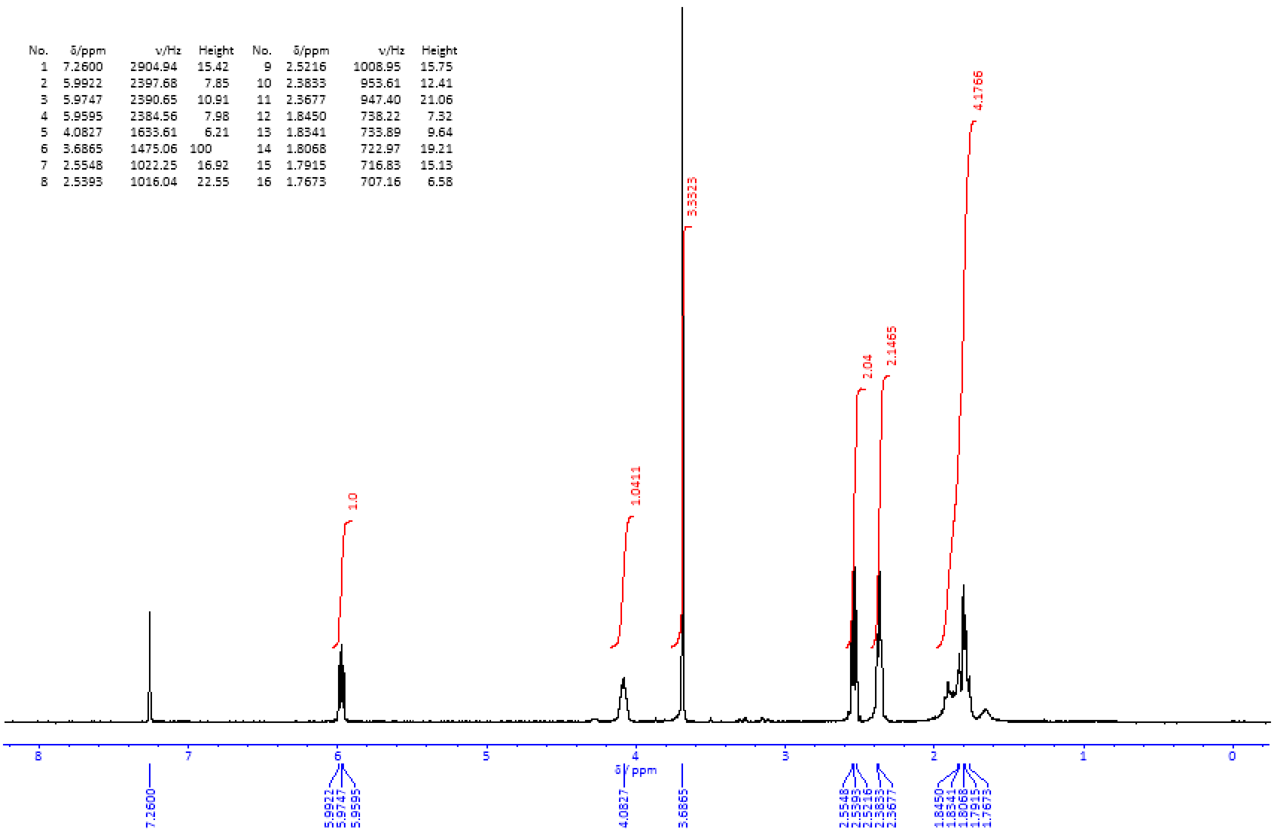


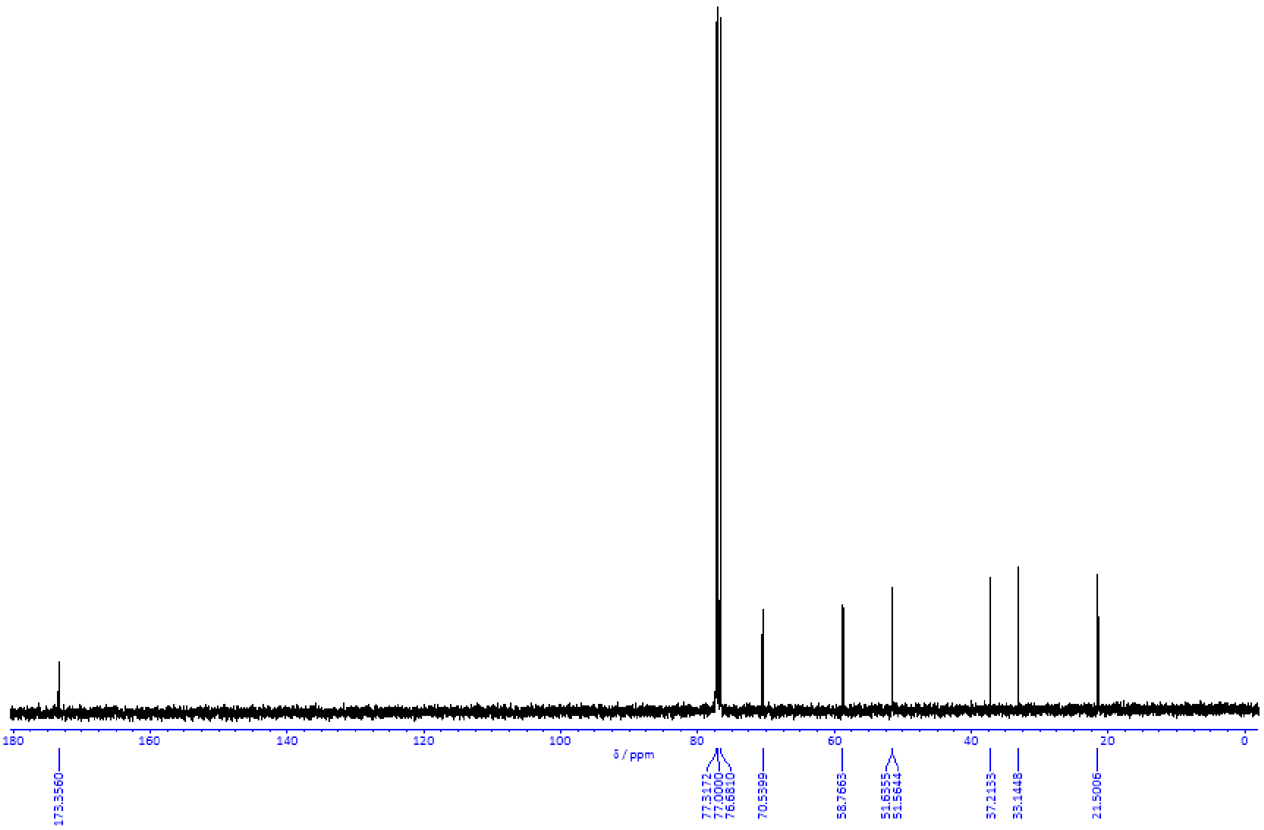


**Figure S7.** $^{1}$H and $^{13}$C NMR spectra of ethyl 5,7,7-trichloroheptanoate:

## After Mixing

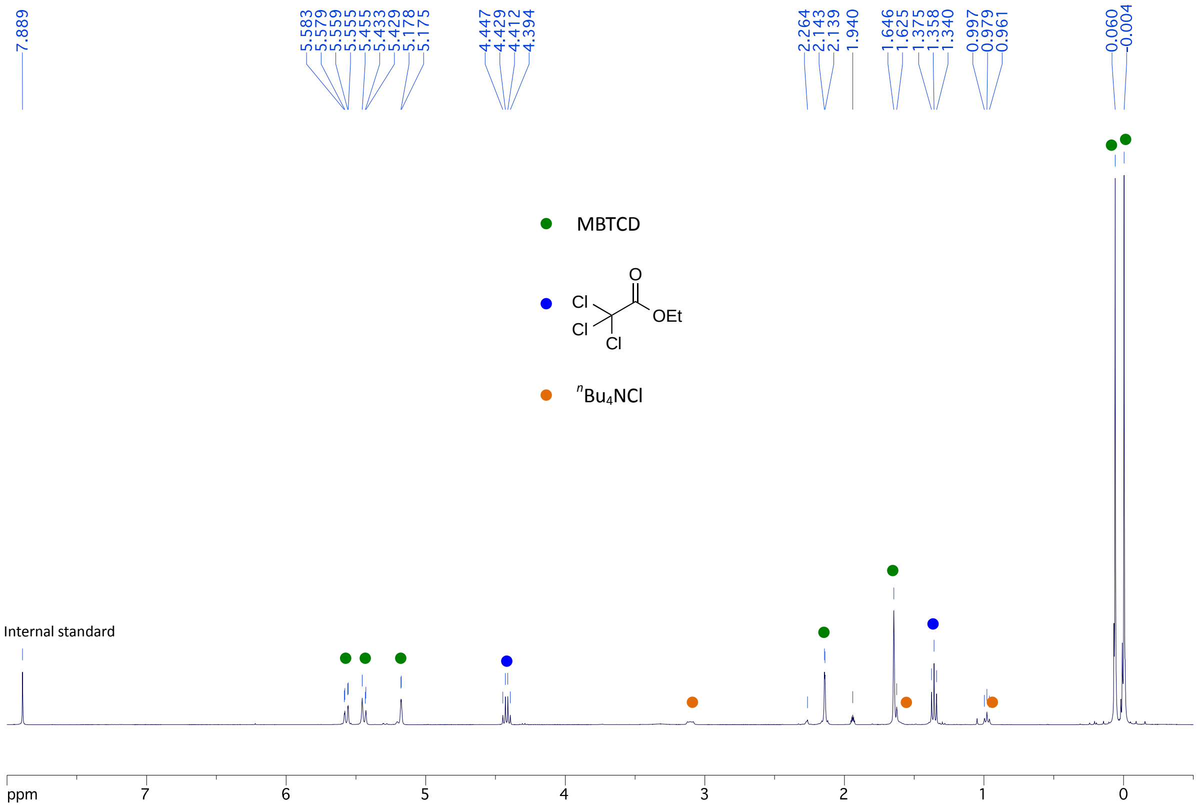


## After heating at 80 °C for 24 h

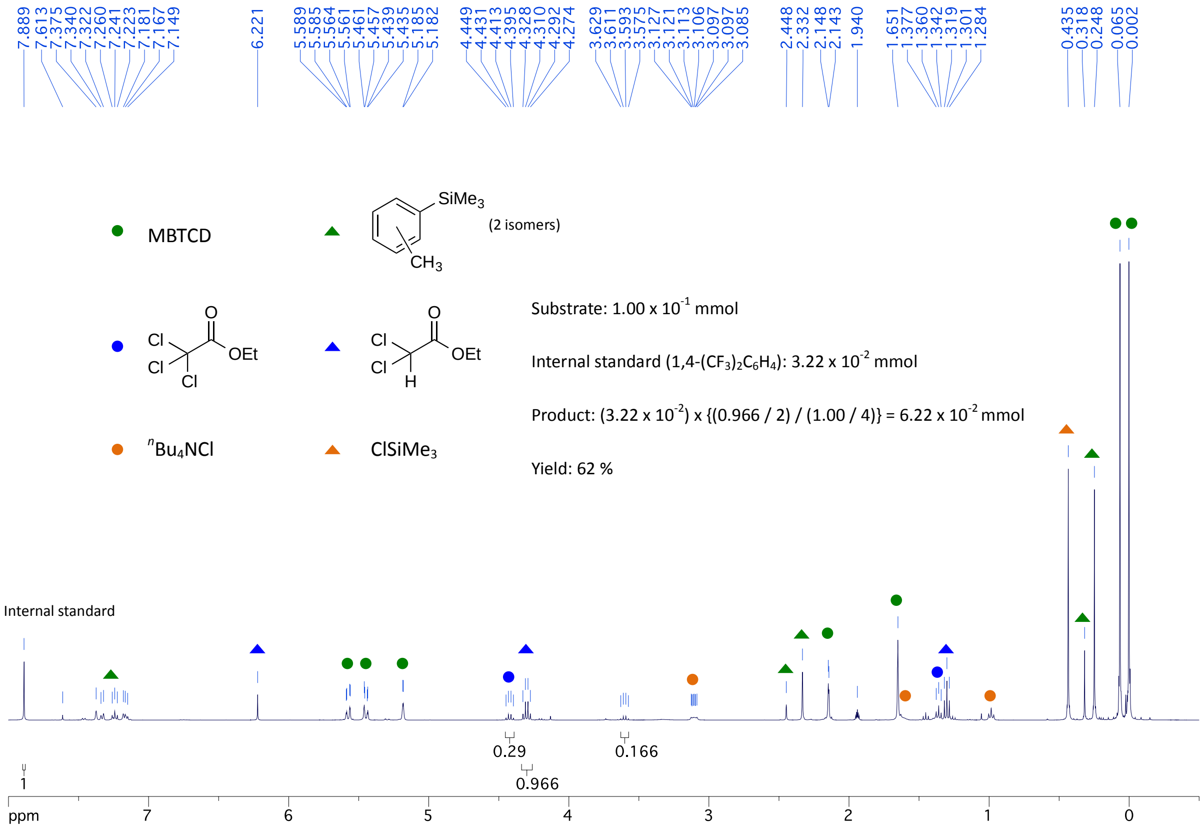


**Figure S8.** $^1$H NMR spectrum of hydrodehalogenation reaction for run 4 in Table 2:

After Mixing

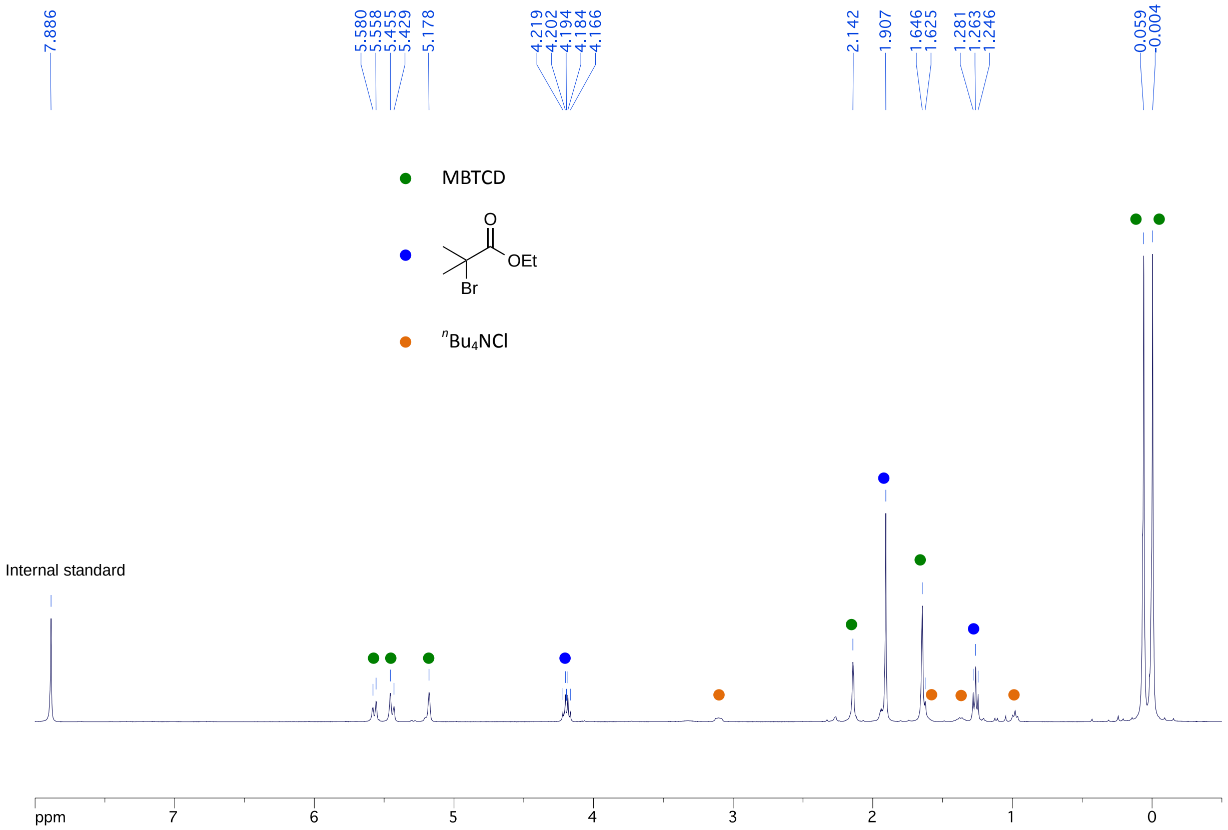


After heating at 80 °C for 24 h

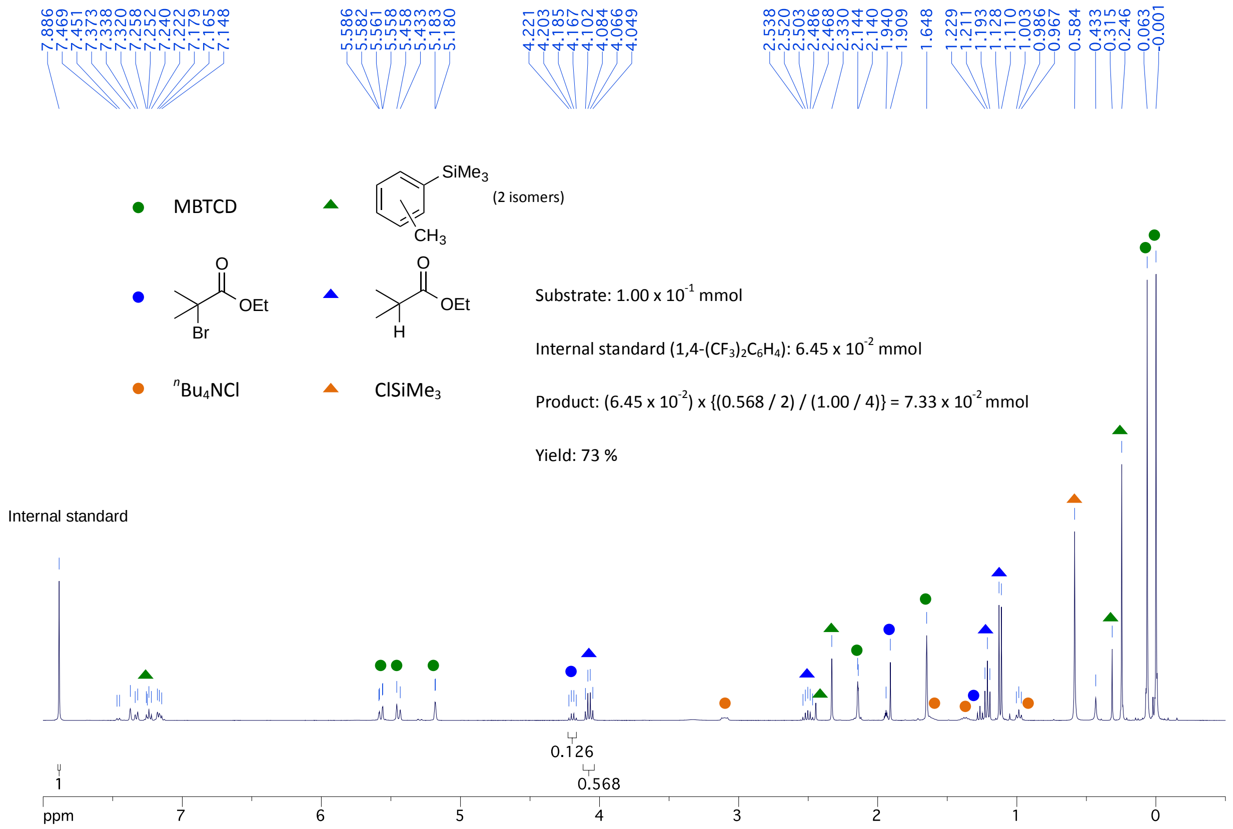


**Figure S9.** $^{1}$H NMR spectrum of hydrodehalogenation reaction for run 5 in Table 2:

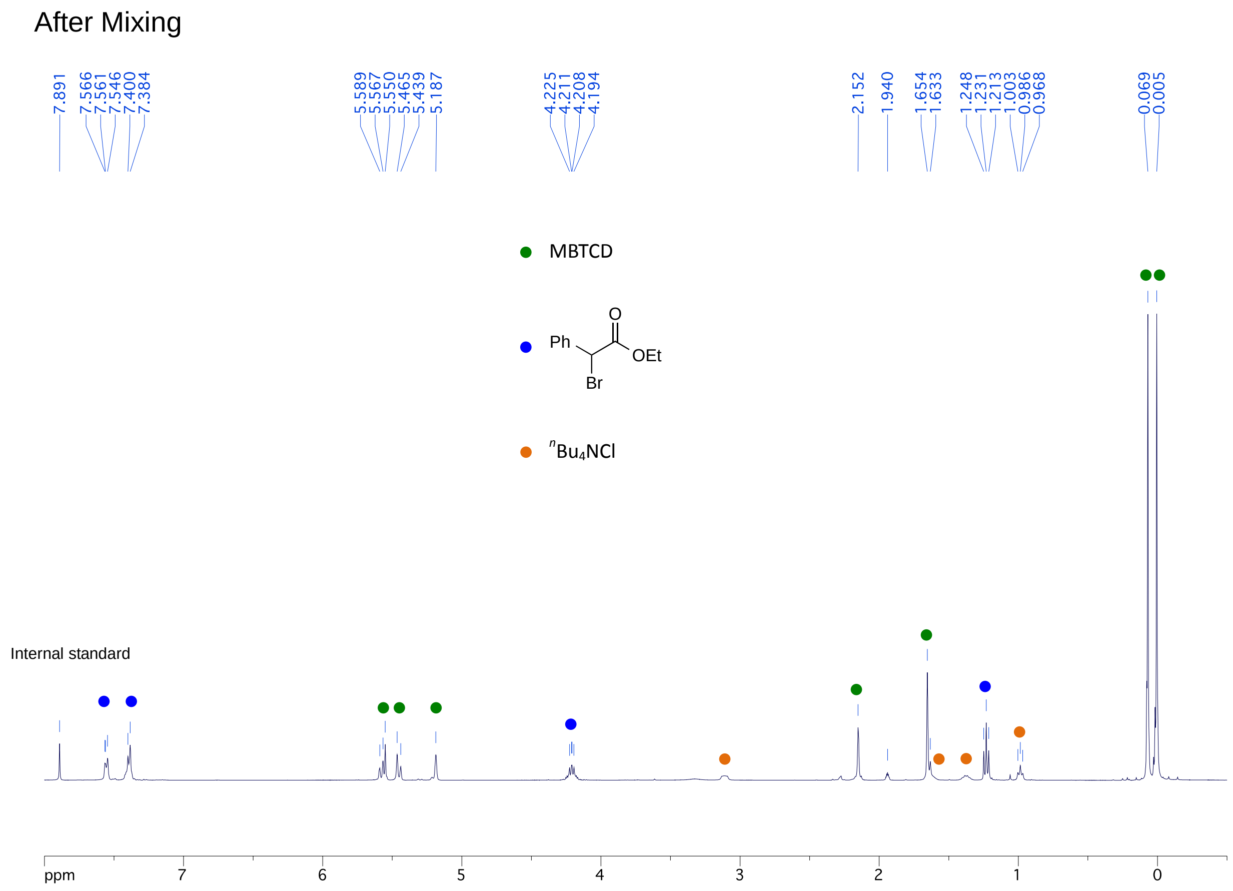


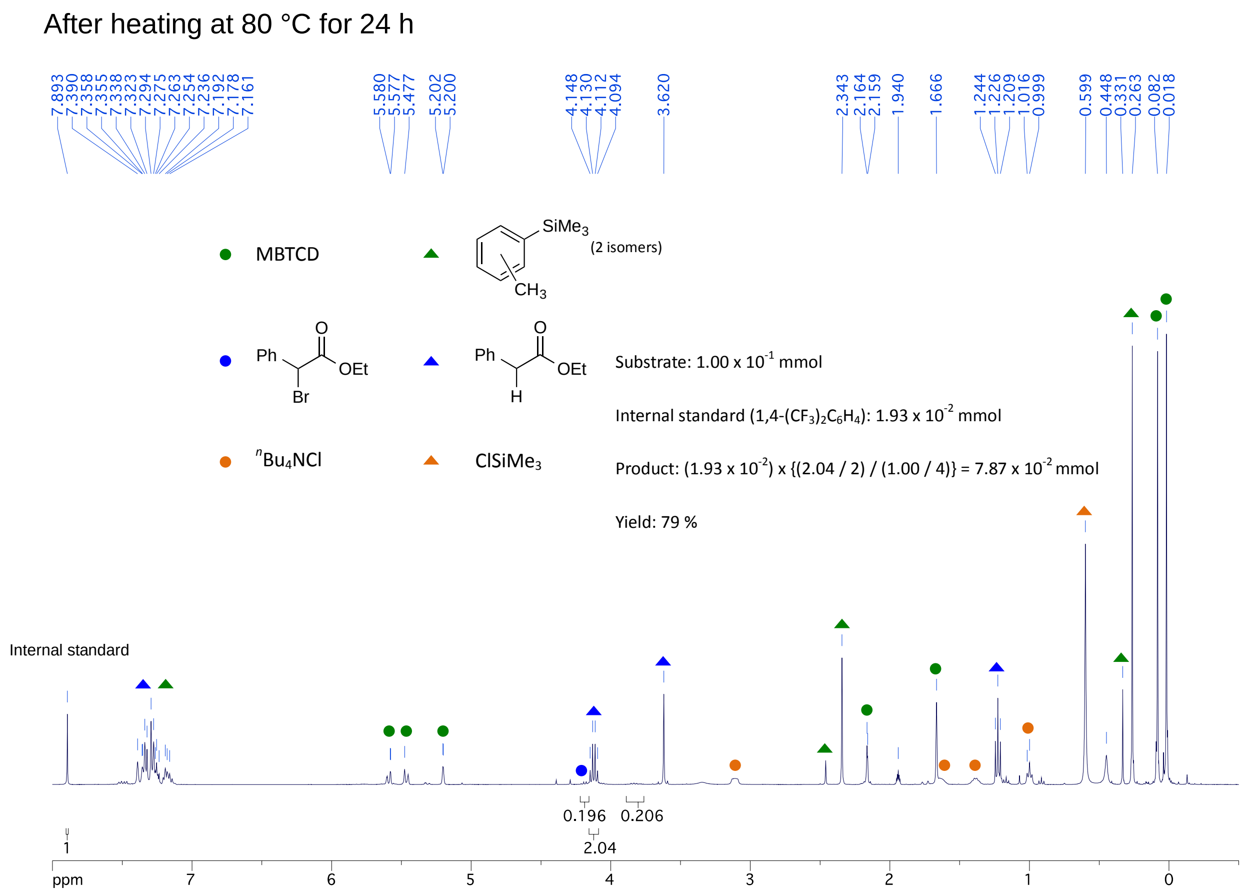


**Figure S10.** $^{1}$H NMR spectrum of hydrodehalogenation reaction for run 6 in Table 2:

## After Mixing

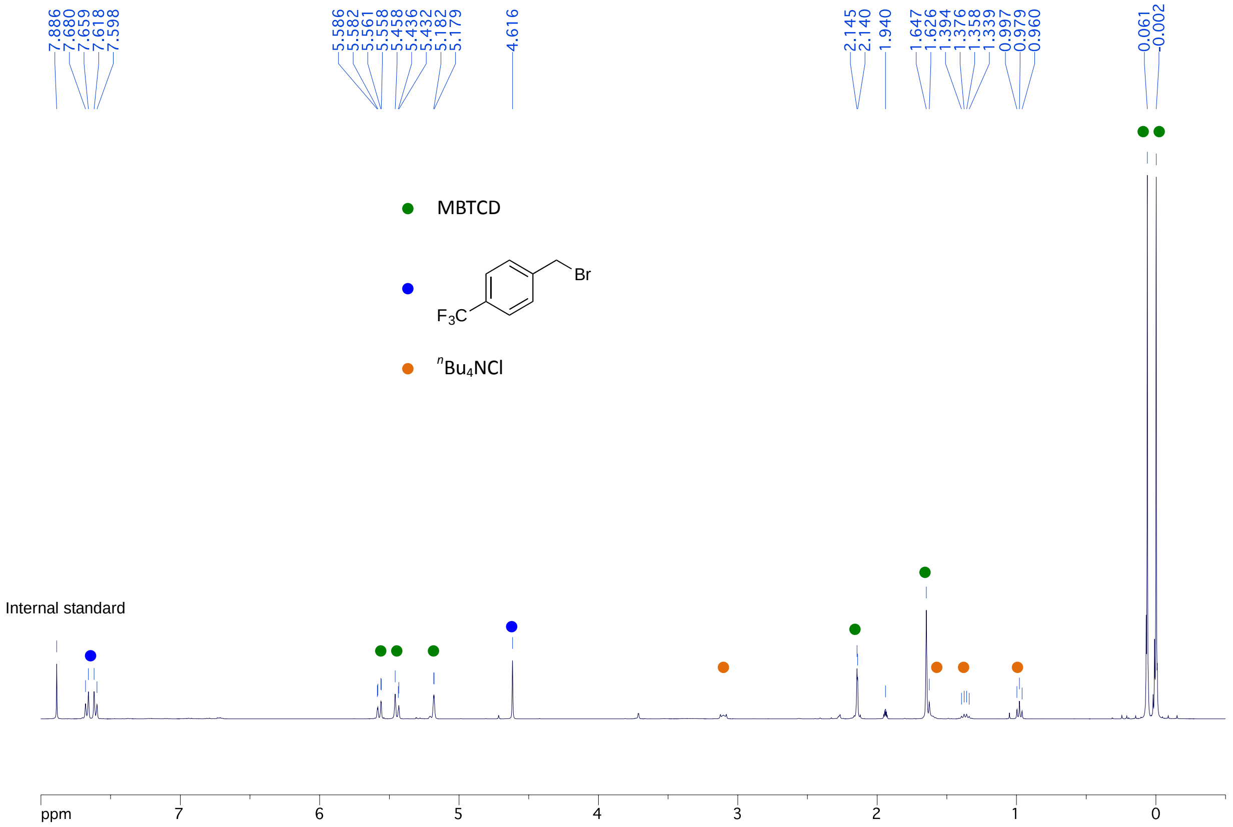

## After heating at 80 °C for 24 h

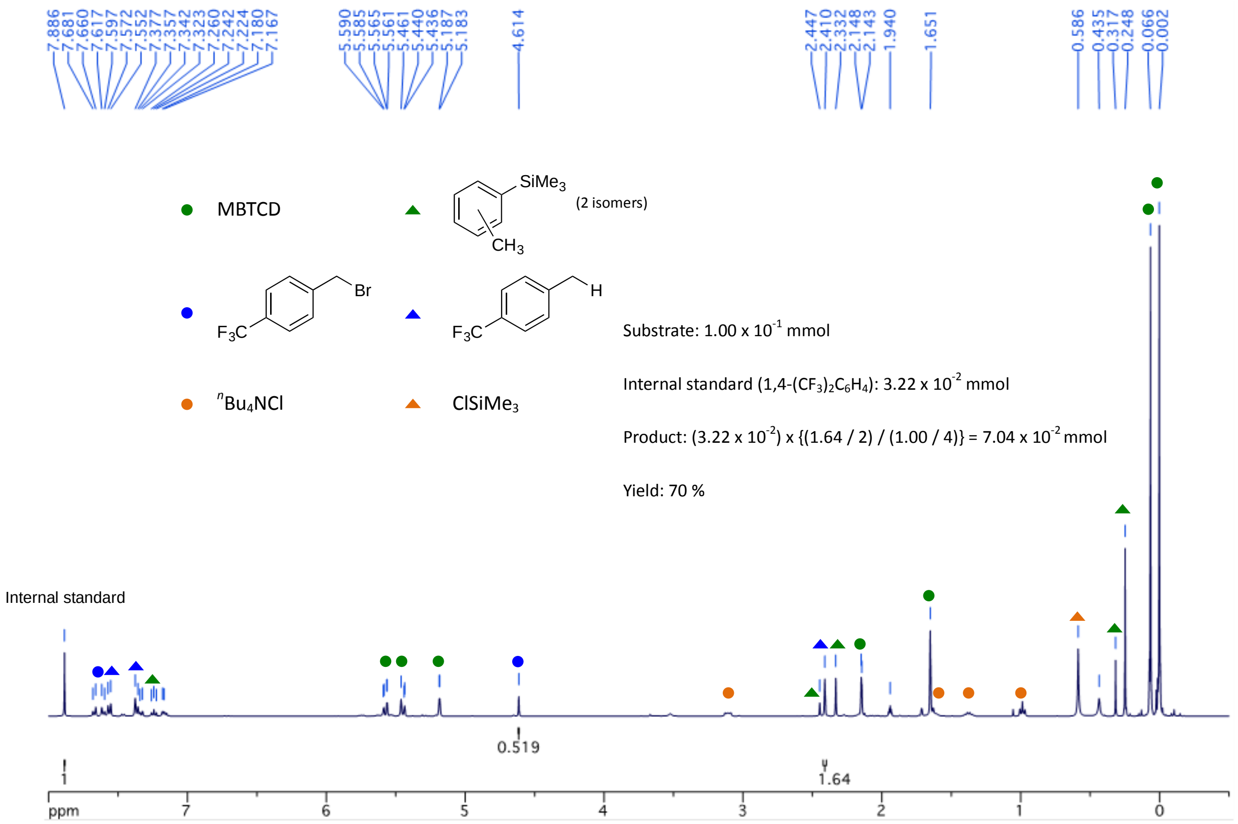


**Figure S11.** $^{1}$H NMR spectrum of hydrodehalogenation reaction for run 7 in Table 2:

## 3. Kinetic Study for Hydrodehalogenation Reaction.

In a glovebox under argon, catalyst, 1,1,1,3-tetrachlorppropane ([A], initial concentration = $[A]_0$), 1,4-bis(trifluoromethyl)benzene as an internal standard, and $CD_3CN$ (0.5 mL) were added to a J-young capped NMR tube. Yield of the product and consumption of the substrate were determined by integral ratios of $^1H$ NMR signals of 1,4-bis(trifluoromethyl)benzene, 1,1,1,3-tetrachlorppropane, and $Cl_2C\mathit{H}CH_2CH_2Cl$. A deuterium-labeling experiment by using MBTCD-$d_8$ was carried out to obtain a KIE value (1.71)

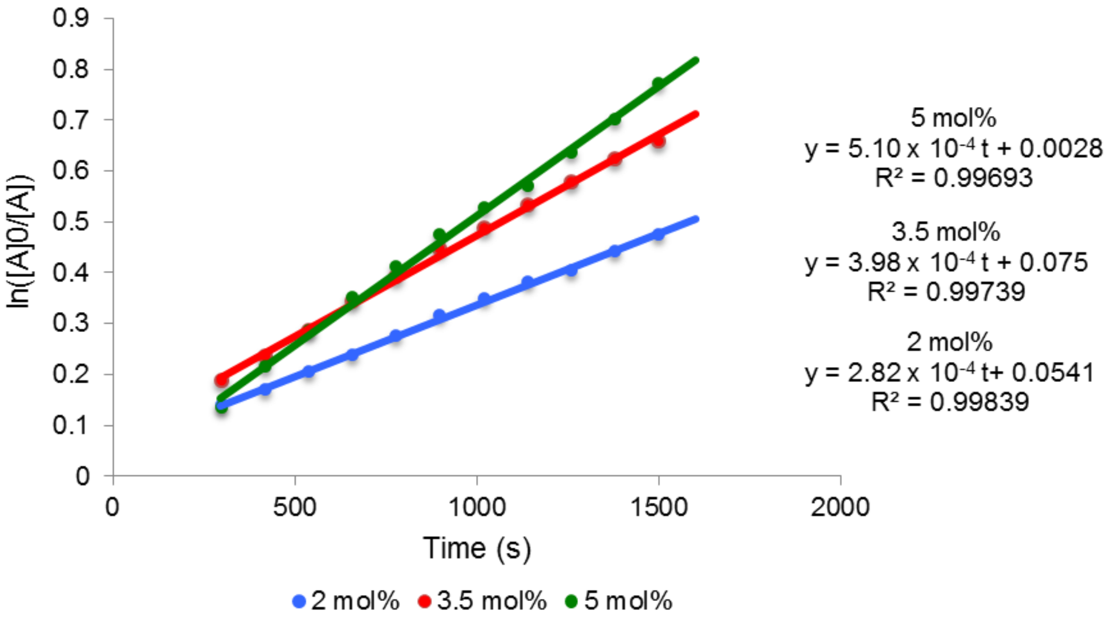


**Figure S12.** First-order plot on substrate consumption for different catalyst loading (reaction at 60 °C, MBTCD = 1.2 equiv in $CD_3CN$).

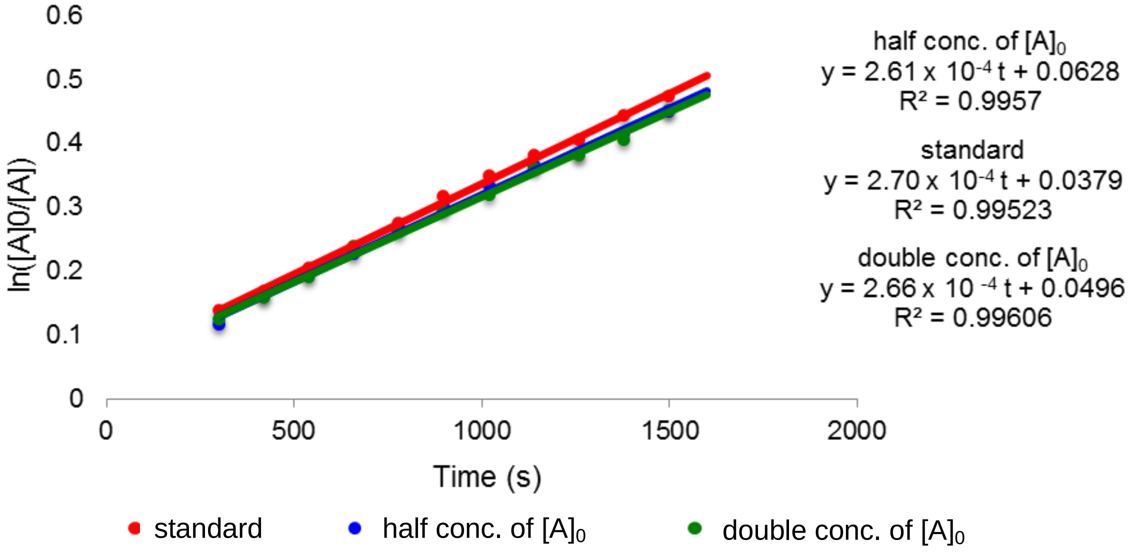


**Figure S13.** First-order plot on substrate consumption for different substrate concentration (reaction at 60 °C, cat. = 2 mol%, MBTCD = 1.2 equiv in $CD_3CN$).

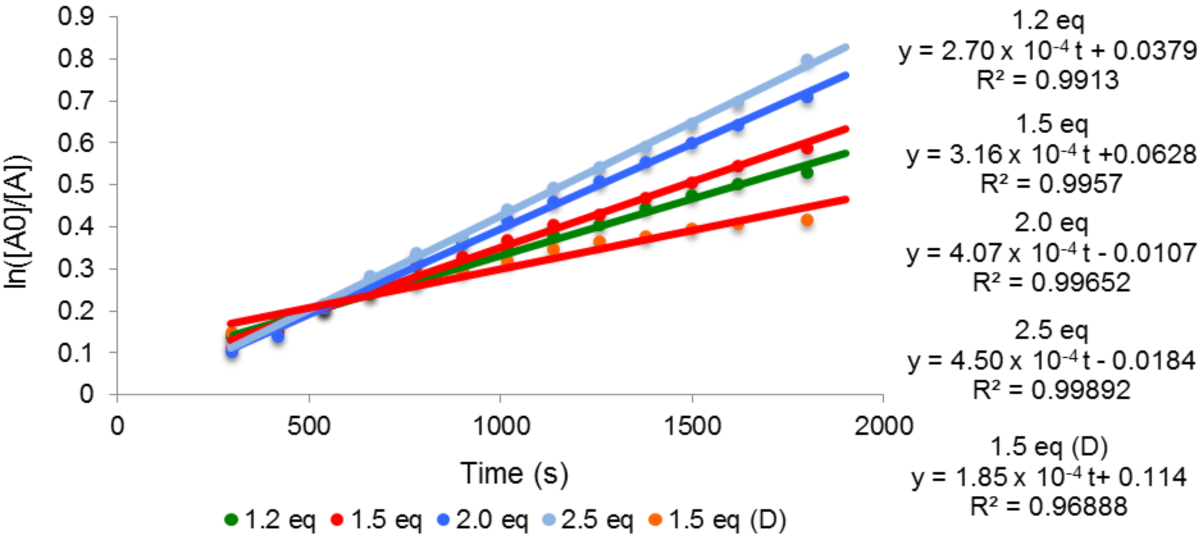


**Figure S14.** First-order plot on substrate consumption for different MBTCD concentration (reaction at 60 °C, cat. = 2 mol% in $CD_3CN$). For determining the KIE value, MBTCD-$d_8$ (1.5 equiv) was used.

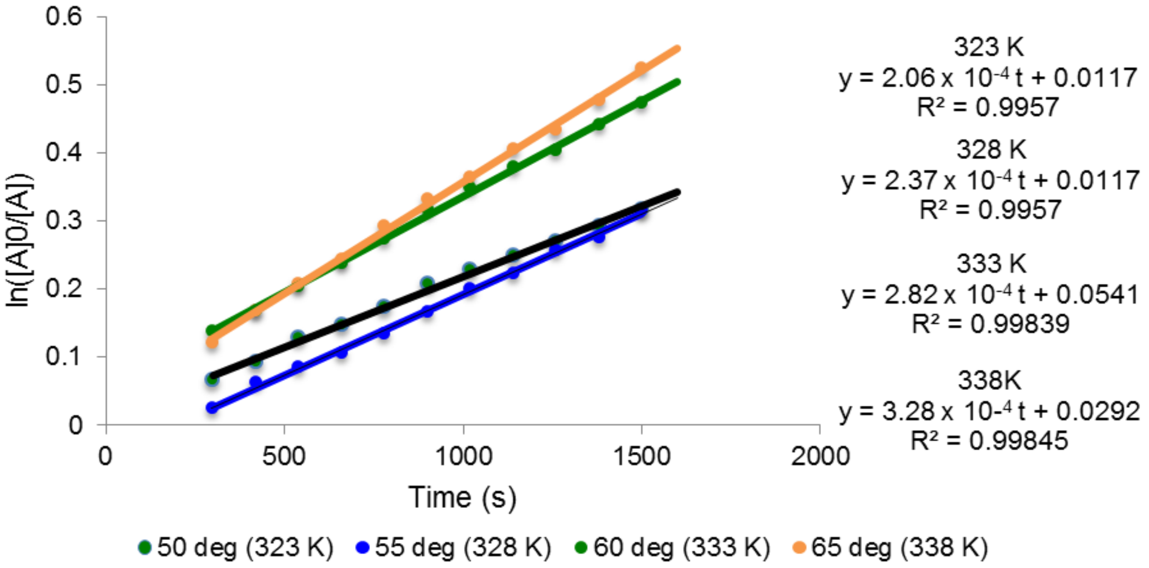


**Figure S15.** First-order plot on substrate consumption at four different reaction temperature (cat. = 2 mol%, MBTCD = 1.2 equiv in $CD_3CN$).

## 4. Molecular Structure of Dinuclear Molybdenum Complexes.

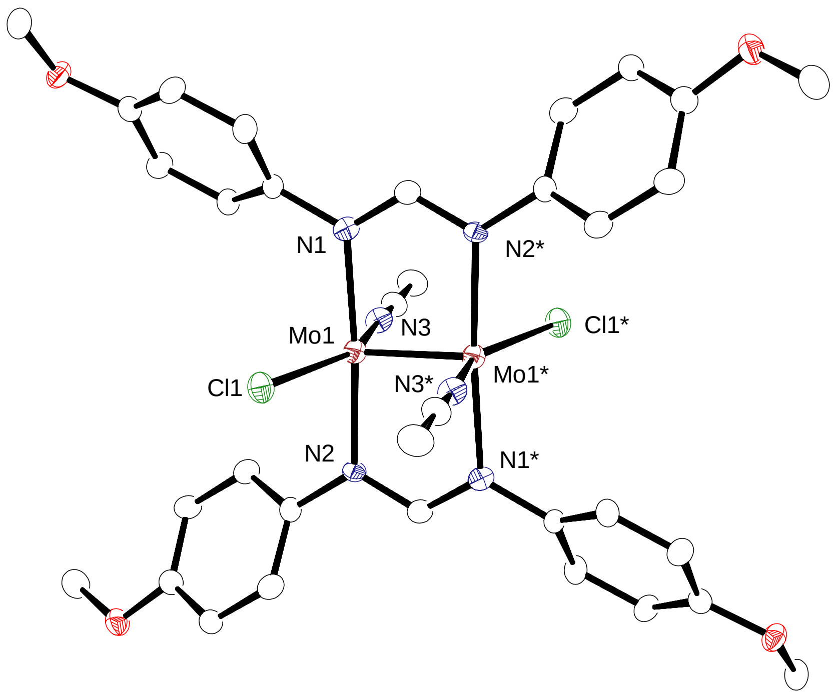


**Figure S16.** ORTEP drawing of complex **7a**. Hydrogen atoms are omitted for clarity.

**Table S1.** Selected bond lengths (Å) of complex **7a**.

| | | | | | |
|---|---|---|---|---|---|
| Mo1 | Mo1* | 2.1227(8) | Mo1 | Cl1 | 2.4395(19) |
| Mo1 | N1 | 2.137(6) | Mo1 | N2 | 2.153(6) |
| Mo1 | N3 | 2.141(7) | | | |

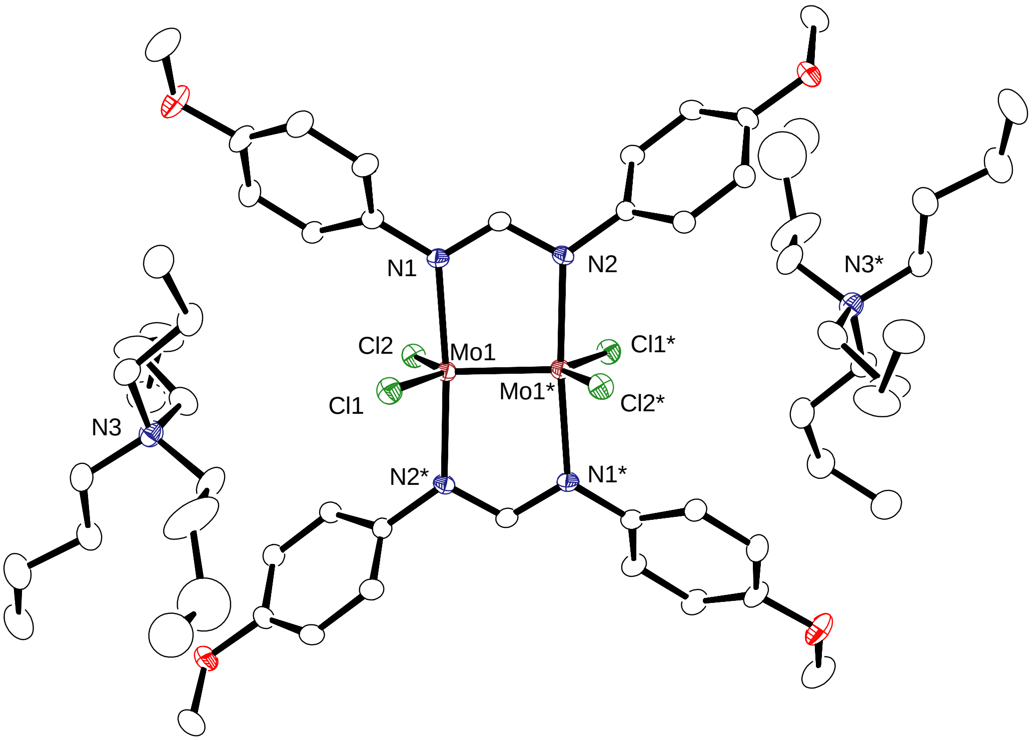


**Figure S17.** ORTEP drawing of complex **9a**. Hydrogen atoms are omitted for clarity.

**Table S2.** Selected bond lengths (Å) of complex **9a**.

| | | | | | |
|---|---|---|---|---|---|
| Mo1 | Mo1* | 2.1153(6) | Mo1 | Cl1 | 2.4578(12) |
| Mo1 | Cl2 | 2.4639(12) | Mo1 | N1 | 2.151(5) |
| Mo1 | N2* | 2.147(5) | | | |

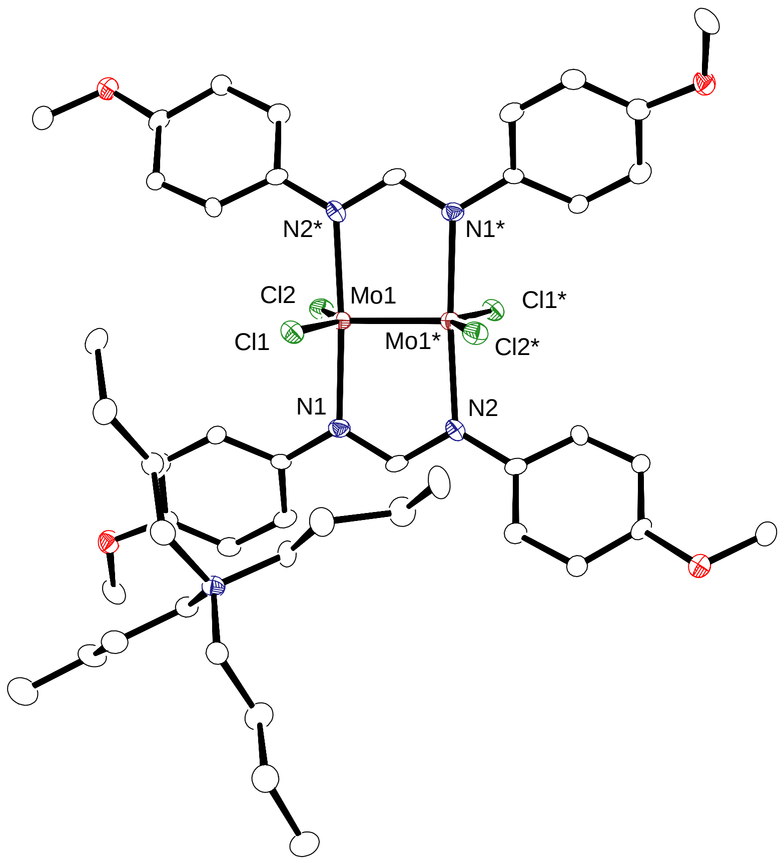


**Figure S18.** ORTEP drawing of complex **10a**. Hydrogen atoms are omitted for clarity.

**Table S3.** Selected bond lengths (Å) of complex **10a**.

| | | | | | |
|---|---|---|---|---|---|
| Mo1 | $Mo1^*$ | 2.1458(9) | Mo1 | Cl1 | 2.396(2) |
| Mo1 | Cl2 | 2.375(3) | Mo1 | N1 | 2.130(6) |
| Mo1 | $N2^*$ | 2.138(7) | | | |

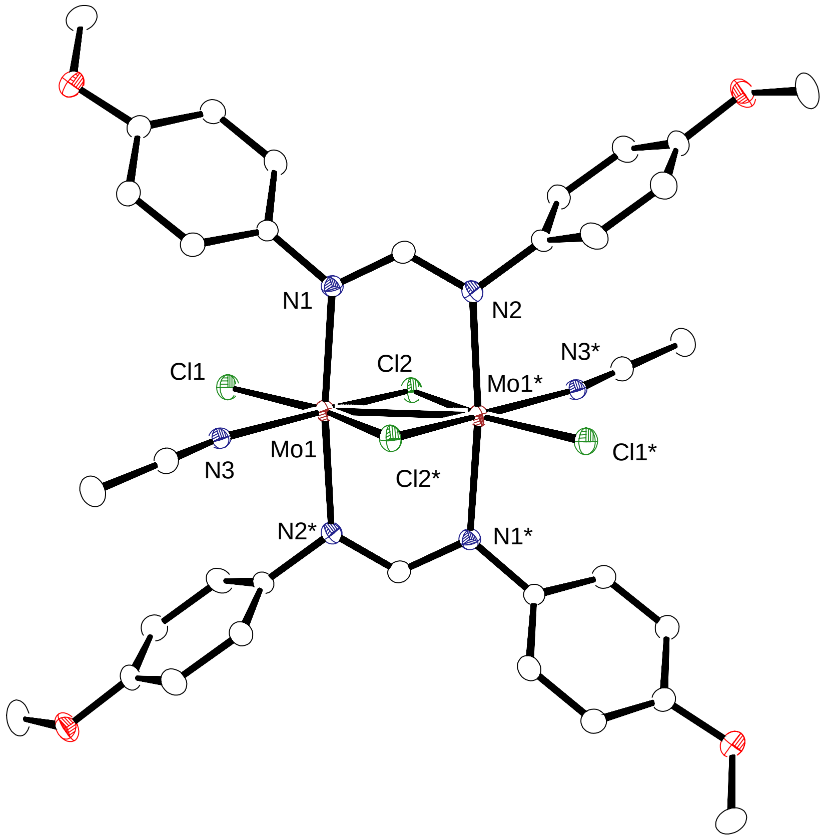


**Figure S19.** ORTEP drawing of complex **11a**. Hydrogen atoms are omitted for clarity.

**Table S4.** Selected bond lengths (Å) of complex **11a**.

| | | | | | |
|---|---|---|---|---|---|
| Mo1 | Mo1* | 2.5971(10) | Mo1 | Cl1 | 2.4171(15) |
| Mo1 | Cl2 | 2.4200(14) | Mo1 | Cl2* | 2.4099(14) |
| Mo1 | N1 | 2.112(4) | Mo1 | N2 | 2.139(4) |
| Mo1 | N3 | 2.186(4) | | | |

**Table S5.** Crystal Data and Data Collection Parameters.

| | **7a** | **9a** |
|---|---|---|
| CCDC No. | 1046579 | 1046580 |
| empirical formula | $C_{34}H_{36}Cl_2Mo_2N_6O_4$ | $C_{62}H_{102}Cl_4Mo_2N_6O_4$ |
| formula weight | 855.48 | 1329.18 |
| crystal system | monoclinic | monoclinic |
| space group | $P2_1/c$ (No. 14) | $P2_1/n$ (No. 14) |
| $a$, Å | 12.536(2) | 12.6064(14) |
| $b$, Å | 12.3769(18) | 14.1061(14) |
| $c$, Å | 13.069(3) | 19.183(2) |
| $\alpha$, deg. | - | - |
| $\beta$, deg. | 115.499(7) | 101.372(6) |
| $\gamma$, deg. | - | - |
| $V$, Å$^3$ | 1830.1(6) | 3344.4(6) |
| $Z$ | 2 | 2 |
| $D_{calcd}$, g/cm$^3$ | 1.552 | 1.320 |
| $\mu$ [Mo-$K\alpha$], mm$^{-1}$ | 0.876 | 0.582 |
| $T$, K | 113(2) | 113(2) |
| crystal size, mm | 0.12 x 0.07 x 0.07 | 0.23 x 0.17 x 0.10 |
| $\theta$ range for data collection (deg.) | 5.74 to 27.42 | 4.98 to 27.47 |
| no. of reflections measured | 16835 | 31317 |
| unique data ($R_{int}$) | 4103 (0.0677) | 7530 (0.0660) |
| data / restraints / parameters | 4103 / 0 / 217 | 7530 / 0 / 342 |
| $R1$ ($I > 2.0\sigma(I)$) | 0.0918 | 0.0783 |
| $wR2$ ($I > 2.0\sigma(I)$) | 0.2248 | 0.1652 |
| $R1$ (all data) | 0.1061 | 0.0877 |
| $wR2$ (all data) | 0.2441 | 0.1724 |
| GOF on $F^2$ | 1.174 | 1.207 |
| $\Delta\rho$, e Å$^{-3}$ | 3.26, -2.03 | 1.470, -0.870 |

a) $R1 = (\Sigma||Fo| - |Fc||)/(\Sigma|Fo|)$ b) $wR2 = [\{\Sigma w(Fo^2\text{-}Fc^2)^2\}/\{\Sigma w(Fo^4)\}]^{1/2}$

**Table S5.** Crystal Data and Data Collection Parameters (Continued).

| | **10a** | **11a** |
|---|---|---|
| CCDC No. | 1046581 | 1046582 |
| empirical formula | $C_{48}H_{69}Cl_4Mo_2N_6O_4$ | $C_{34}H_{36}Cl_4Mo_2N_6O_4$ |
| formula weight | 1127.80 | 926.39 |
| crystal system | triclinic | monoclinic |
| space group | $P\bar{1}$ (No. 2) | $P2_1/c$ (No. 14) |
| $a$, Å | 12.142(4) | 11.752(5) |
| $b$, Å | 13.615(4) | 15.014(7) |
| $c$, Å | 17.274(6) | 11.140(5) |
| $\alpha$, deg. | 78.896(9) | - |
| $\beta$, deg. | 84.467(11) | 109.276(3) |
| $\gamma$, deg. | 69.112(8) | - |
| $V$, $Å^3$ | 2616.8(14) | 1855.4(14) |
| $Z$ | 2 | 2 |
| $D_{calcd}$, $g/cm^3$ | 1.431 | 1.658 |
| $\mu$ [Mo-$K\alpha$], $mm^{-1}$ | 0.730 | 1.010 |
| $T$, K | 113(2) | 113(2) |
| crystal size, mm | 0.17 x 0.15 x 0.05 | 0.21 x 0.20 x 0.13 |
| $\theta$ range for data collection (deg.) | 4.82 to 24.50 | 5.98 to 27.50 |
| no. of reflections measured | 19667 | 17056 |
| unique data ($R_{int}$) | 8509 (0.0426) | 4159 (0.0463) |
| data / restraints / parameters | 8509 / 0 / 577 | 4159 / 0 / 226 |
| $R1$ ($I > 2.0\sigma(I)$) | 0.0819 | 0.0571 |
| $wR2$ ($I > 2.0\sigma(I)$) | 0.2007 | 0.1151 |
| $R1$ (all data) | 0.0971 | 0.0619 |
| $wR2$ (all data) | 0.2250 | 0.1184 |
| GOF on $F^2$ | 1.195 | 1.215 |
| $\Delta\rho$, e $Å^{-3}$ | 3.04, -1.22 | 1.57, -0.79 |

a) $R1 = (\Sigma||Fo| - |Fc||)/(\Sigma|Fo|)$ b) $wR2 = [\{\Sigma w(Fo^2-Fc^2)^2\}/\{\Sigma w(Fo^4)\}]^{1/2}$

## 5. References.


S1) Laguerre, M.; Dunogues, J.; Calas, R.; Duffaut, N. *J. Organomet. Chem.* **1976**, *112*, 49.

S2) Brignole, A. B.; Cotton, F. A. *Inorg. Synth.* **1972**, *13*, 81.

S3) Wu, Y.-Y.; Chen, J.-D.; Liou, L.-S.; Wang, J.-C. *Inorg. Chim. Acta* **2002**, *336*, 71.

S4) Cotton, F. A.; Liu, C. Y.; Murillo, C. A. *Inorg. Chem.* **2004**, *43*, 2267.

S5) Cotton, F. A.; Liu, C. Y.; Murillo, C. A.; Villagrán, D.; Wang, X. *J. Am. Chem. Soc.* **2003**, *125*, 13564.

S6) Lin, C.; Protasiewicz, J. D.; Smith, E. T.; Ren, T. *Inorg. Chem.* **1996**, *35*, 6422.

S7) Pangborn, A. B.; Giardello, M. A.; Grubbs, R. H.; Rosen, R. K.; Timmers, F. J. *Organometallics* **1996**, *15*, 1518.

S8) Cotton, F. A.; Liu, C. Y.; Murillo, C. A.; Wang, X. *Chem. Commun.* **2003**, 2190.

S9) Altomare, A.; Cascarano, G.; Giacovazzo, C.; Guagliardi, A.; Burla, M.; Polidori, G.; Camalli, M. *J. Appl. Cryst.* **1994**, *27*, 435.

S10) Sheldrick, G.M. *Acta Cryst.* **2008**, *A64*, 112.

S11) Farrugia, L. J. *J. Appl. Cryst.* **1999**, *32*, 837.

S12) Asahara, T.; Seno, M.; Wu, C.-C, Kogyo Kagaku Zasshi, **1969**, *72*, 1818.

S13) Kano, H.; Matsumoto H.; Nagai, Y. *J. Organomet. Chem.* **1978**, *148*, 276.
